# From nutritional intervention to immune modulation: a multi-database bibliometric and topic modeling study of vitamin D in inflammatory bowel disease

**DOI:** 10.3389/fimmu.2026.1845767

**Published:** 2026-05-29

**Authors:** Kun Wang, Xingyu Zhu, Xinyu Ju, Meiqi Zhou, Guoming Shen

**Affiliations:** 1 School of Integrated Chinese and Western Medicine, Anhui University of Chinese Medicine, Hefei, Anhui, China; 2 Department of Laboratory Medicine, The First Hospital of China Medical University, Shenyang, China; 3 Department of Biochemistry and Molecular Biology, School of Laboratory Medicine, Bengbu Medical University, Bengbu, Anhui, China; 4 Anhui Province Key Laboratory of Meridian Viscera Correlation ship, Anhui University of Chinese Medicine, Hefei, Anhui, China

**Keywords:** bibliometrics, emerging frontiers, inflammatory bowel disease, research trends, vitamin D

## Abstract

**Background:**

Inflammatory bowel disease (IBD) is a chronic condition characterized by recurrent inflammatory episodes in the gastrointestinal tract. Conventional therapies, including biologics, corticosteroids, and immunosuppressive drugs, can effectively alleviate disease activity. However, their utility is often limited by adverse effects. Vitamin D plays a role in modulating immune function, intestinal barrier integrity, and the gut microbiota. It has emerged as a promising adjunctive therapy for IBD.

**Objective:**

To map the research landscape of vitamin D in IBD using bibliometric analysis, focusing on knowledge evolution, core themes, and emerging trends.

**Methods:**

Publications from 2006 to 2025 were collected by searching the Web of Science Core Collection (WoSCC) and Scopus databases. After removing duplicates, 2,659 articles and reviews were obtained for analysis. Networks were constructed using CiteSpace, VOSviewer, R, and Python. PubMed clinical trials were included for complementary analysis, and BERTopic was applied to identify latent topics and their temporal dynamics.

**Results:**

A total of 2,659 publications spanning 970 journals were identified, with a steady increase in output. The USA was the leading contributor and also demonstrated strong international collaboration. Ananthakrishnan Ashwin N. was the most influential author, and Inflammatory Bowel Diseases was the journal with the leading journal. Core themes included vitamin D deficiency, gut microbiota, inflammatory response, diet and nutrition. The focus has shifted toward gut microbiota, immune regulation, micronutrients, and evidence-based approaches. Furthermore, BERTopic modeling identified 12 latent topics, with increasing emphasis on gut microbiota, fecal microbiota transplant, and nutritional deficiencies.

**Conclusion:**

This bibliometric analysis provides a concise, data-driven overview of research on vitamin D in IBD. The results highlighting its structure, evolution, and emerging trends, and informing future mechanistic and translational research.

## 1 Introduction

Inflammatory bowel disease (IBD) is a chronic condition characterized by recurrent episodes of inflammation in the gastrointestinal tract. It comprises Crohn’s disease (CD) and ulcerative colitis (UC). UC affects the rectum and colon, while CD can affect any part from the mouth to the anus (1). The clinical manifestations of IBD involve diarrhea, weight loss, nocturnal defecation, pain, and fatigue (2). Although the pathogenesis of IBD is not fully understood, it is closely associated with environmental influences, genetic susceptibility, intestinal microbiota dysbiosis, impaired gut barrier function, and abnormal immune responses (3, 4). Conventional therapies primarily include biologics, glucocorticoids, and immunosuppressants. However, these treatments can cause adverse reactions, including systemic immunosuppression, insufficient targeting of intestinal inflammation, impairment of renal and hepatic function, and abnormal bone metabolism (5). Therefore, seeking safer and more effective therapeutic strategies is of significant clinical importance.

Vitamin D deficiency plays an important role in the development and progression of IBD (6, 7). Vitamin D, a fat-soluble steroid hormone, not only maintains normal immune function but also preserves intestinal barrier integrity and gut microbiota steady state, offering a promising therapeutic approach for IBD (8). Studies have shown that vitamin D can modulate immune responses, inflammation, and antioxidant activity (9). It can improve IBD through multiple pathways, including maintaining intestinal epithelial integrity, regulating gut microbiota balance, and modulating immune responses (10, 11). As a result, vitamin D-based approaches for improving IBD have emerged as a promising strategy (12). This theoretical foundation has increased research interest in this field and prompted systematic evaluations of its research trends.

Bibliometrics is a method that conducts quantitative and qualitative analysis of literature in a specific field, enabling the intuitive revelation of its knowledge structure, research trends, collaboration networks, and emerging hotspots (13, 14). This study used bibliometric methods to perform an overview of countries, institutions, journals, authors, keywords, and cited references in the field of vitamin D and IBD. In particular, integrating Web of Science Core Collection (WoSCC) and Scopus enhanced the comprehensiveness and reliability of the results. Randomized controlled trials from PubMed were used as supplementary evidence to identify clinical trials and translational medicine research, to offset the limited coverage of clinical studies in WoSCC and Scopus. This research contributes to an understanding of the knowledge structure and research hotspots of vitamin D in IBD, and provides a data-driven foundation for guiding future mechanism research and clinical translation.

## 2 Methods

### 2.1 Data sources and data cleaning

**Figure 1:**
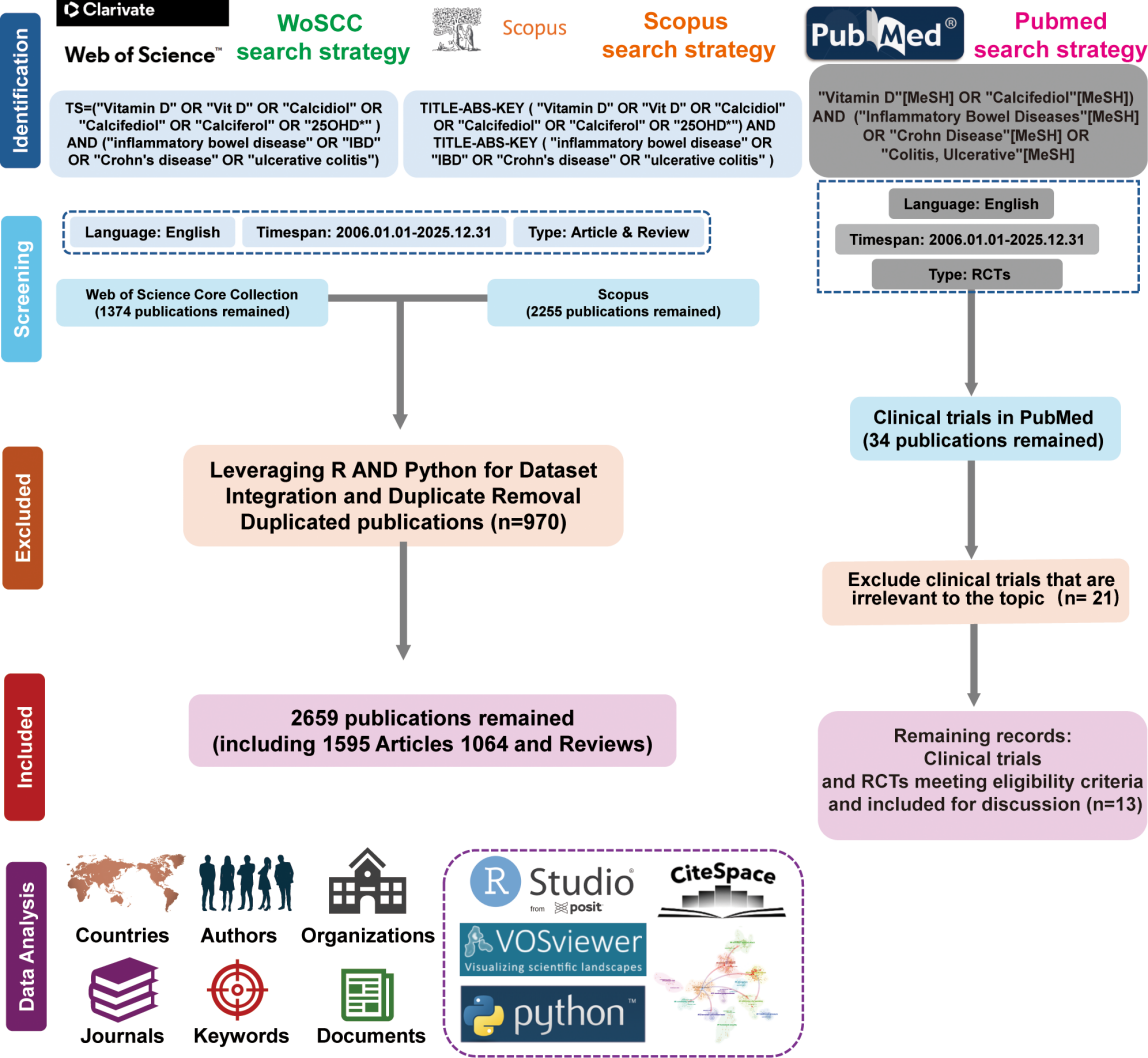
Flowchart of literature screening and dataset integration. The workflow illustrates the retrieval and processing of publications from WoSCC (n = 1,374) and Scopus (n = 2,255). Non-English articles and non-research/review records were excluded, and publication dates were restricted to January 2006–December 2025. Duplicate records (n = 970) were removed using Python and R, resulting in 2,659 publications (1,595 articles and 1,064 reviews) for analysis. Bibliometric analyses were subsequently conducted using CiteSpace, VOSviewer, Python, and R to characterize research trends, collaboration networks, and thematic evolution.

The data for this study were obtained from two academic databases, WoSCC and Scopus, which improves the robustness of the results. The search period spanned from January 1, 2006, to December 31, 2025, and only English-language publications and the document types restricted to Article and Review. The search strategy used Boolean operators to combine subject terms for vitamin D and IBD. The detailed search queries were as follows: TS = (“Vitamin D” OR “Vit D” OR “Calcidiol” OR “Calcifediol” OR “Calciferol” OR “25OHD*”) AND (“inflammatory bowel disease” OR “IBD” OR “Crohn’s disease” OR “ulcerative colitis”) for WoSCC, and TITLE-ABS-KEY (“Vitamin D” OR “Vit D” OR “Calcidiol” OR “Calcifediol” OR “Calciferol” OR “25OHD*”) AND TITLE-ABS-KEY (“inflammatory bowel disease” OR “IBD” OR “Crohn’s disease” OR “ulcerative colitis”) for Scopus (**Supplementary Table 1**). Following the search, the initial results included 1,374 records from WoSCC and 2,255 records from Scopus. To ensure a unified database, data integration and deduplication were performed using R-Bibliometrix and Python scripts. By aligning metadata such as article titles, authors, and publication years, 970 duplicate articles were identified and removed (15). A total of 2,659 publications were ultimately included in the analysis, comprising 1,595 research articles and 1,064 review articles (**Figure 1**). In addition, the citation structure of PubMed data lacks several key fields compared with those of WoSCC and Scopus, limiting the applicability of most bibliometric analyses. Therefore, a supplementary analysis of randomized controlled trials in the PubMed database was conducted to identify studies related to clinical trials and translational medicine, thereby compensating for the limited coverage of clinical research in WoSCC and Scopus. The search strategy was: (“Vitamin D” [MeSH] OR “Calcifediol” [MeSH]) AND (“Inflammatory Bowel Diseases” [MeSH] OR “Crohn Disease” [MeSH] OR “Colitis, Ulcerative” [MeSH]) (**Supplementary Table 1**). The publication type was limited to RCTs, covering studies published from 2006 to 2025. Inclusion criteria were: ① Participants (P): Patients diagnosed with IBD. ② Interventions (I): Oral or intramuscular supplementation of vitamin D2 or vitamin D3. ③ Comparators (C): Usual treatments. ④ Outcomes (O): No limitation. ⑤ Study type (S): RCTs. Exclusion criteria were: ① Non-randomized studies, reviews, case reports, abstracts, animal studies. ② Studies without IBD patients or without vitamin D as an intervention. ③ No clear control group. ④ Duplicates or insufficient data. For subsequent analyses, the titles, abstracts, and keywords of all included publications were used as the corpus. During the deduplication process, text standardization was conducted using the disambiguation functions in the bibliometrix package, including the removal of non-alphabetic characters, punctuation, and redundant whitespace, to minimize noise arising from formatting inconsistencies. Duplicate records were identified and eliminated by comparing key bibliographic metadata, including titles, author names, and publication years.

### 2.2 Bibliometric analysis

A suite of bibliometric tools was used to perform a multidimensional quantitative analysis. This analysis included key indicators such as country and regional distribution, collaboration networks, institutional contributions, journal co-occurrence networks, and citation structures. Descriptive statistical analysis, including annual publication counts, identification of core authors, journal distribution, and citation analysis, was conducted using the bibliometrix package (v5.0) in R (v4.4.5). Raw network data were then extracted (16). Collaboration networks for authors, countries, and institutions were constructed with the R bibliometrix package, clustered using the Louvain algorithm, and normalized with the association strength method. To ensure the maps focus on core patterns, only the 50 nodes with the highest association were selected (Number of Nodes = 50), and isolated nodes were removed (Remove Isolated Nodes = Yes). Author, country, and institution networks were constructed using VOSviewer (v1.6.20), employing the Fruchterman–Reingold force-directed algorithm for layout, applying association strength normalization to control for scale effects, and using the Walktrap algorithm for community detection (17). For journal co-citation network analysis in VOSviewer, a cited-source threshold of ≥500 was set, so only sources cited at least 500 times were included. As a result, the co-citation network comprised 38 major journals, each meeting the threshold and serving as a major source in the network. CiteSpace (v6.2.R1) was used for keyword clustering, keyword burst detection, and reference burst analysis. The time slice was defined as one year. Node selection strictly adhered to these parameters (18): G-index (k = 25), LRF = 2.5, L/N = 10, LBY = 5, and e = 1.0. It should be noted that the keyword clustering map was generated by CiteSpace. CiteSpace does not simply retain keywords with “occurrence count > X.” Instead, the default g-index (k = 25) is used as the node selection criterion, which dynamically calculates a threshold based on the keyword frequency distribution within each time slice. The clustering process adopts the log-likelihood ratio (LLR) algorithm to identify and delineate research themes. Each cluster label is automatically generated by the software using the LLR algorithm, selecting terms that best summarize the distinguishing features of the keywords grouped in that cluster. Raw data from some software was imported into a Python (v3.14) environment. This data was then formatted and visually enhanced using libraries such as pandas, matplotlib, seaborn, and networkx.

### 2.3 BERTopic-based topic modeling

To address the limitations of traditional bibliometric methods in semantic depth mining, this study introduces the BERTopic framework for latent topic identification and dynamic evolution analysis. This method generates document embeddings using a pre-trained language model and applies a density-based clustering algorithm to automatically identify semantically coherent topic structures without requiring a predefined number of topics. The overall workflow is as follows: ① Text preprocessing: Standardize the original text by converting it to lowercase, removing non-alphabetic characters and punctuation, and eliminating English stopwords to obtain a clean corpus suitable for embedded computation. ② Document embedding construction: The pre-trained model paraphrase-multilingual-MiniLM-L12-v2 was loaded using SentenceTransformer, and the text was mapped into 384-dimensional semantic vectors. This model has demonstrated robust performance in multilingual semantic similarity evaluations, effectively capturing latent semantic relationships across texts. ③ Dimensionality reduction: The UMAP algorithm is employed for manifold learning and for reducing the dimensionality of the high-dimensional embedding vectors. The parameters are set as n_neighbors = 15, n_components = 5, and min_dist = 0.05. Cosine similarity is used as the distance metric. This approach enhances cluster separability while preserving the local structure of the data. ④ Density-based clustering: Cluster analysis is performed by the HDBSCAN algorithm, with parameters set to min_cluster_size = 25 and min_samples = 10. The cluster selection strategy uses the Excess of Mass (eom) method to identify stable, semantically consistent document clusters. ⑤ Topic representation and interpretation: Representative keywords for each cluster are extracted through the class-based Term Frequency–Inverse Document Frequency (c-TF-IDF) method. This method aggregates intra-cluster word frequencies and weights them with inverse document frequency. It enhances topic distinctiveness and ultimately generates 12 interpretable topic labels. ⑥ Temporal evolution analysis: The topics_over_time module is used to calculate the frequency trends of topics by year. Linear regression is applied for fitting. The trend direction and statistical significance are assessed by calculating R² and *P*-values. *P* < 0.05 is considered significant. To ensure statistical robustness, topics with a time span of less than 5 years are excluded. All BERTopic analyses are performed in a Python (v3.14) environment, using the bertopic, sentence-transformers, umap-learn, hdbscan, scikit-learn, and pygam libraries.

## 3 Results

### 3.1 General characteristics of the publications

This study retrieved 1,374 publications from WoSCC and 2,255 records from Scopus. After deduplication, a total of 2,659 publications (1,595 articles and 1,064 reviews) were retained, distributed across 970 distinct journals. From January 1, 2006, to December 31, 2025, the overall annual publication output showed a steady upward trend, with an average annual growth rate of 6.93%. This growth rate was calculated using the compound annual growth rate (CAGR) formula, which compares the publication counts at the beginning and end of the study period. Overall, this trend shows sustained scholarly interest in this research direction. The average citation count per included article was 49.33, suggesting the field has achieved notable academic impact. Overall, 12,939 authors contributed to the relevant research, of which 157 were single-authored. Each publication had an average of 5.91 co-authors, and 18.2% of the publications involved international collaboration (**Table 1**; **Supplementary Figure 1**). In pubmed, the overall annual publication output showed a steady upward trend, with an average annual growth rate of 6.93%. Additionally, the number of clinical trial publications in PubMed has exhibited a consistent upward trend (**Supplementary Figure 2**).

**Table 1:** Comparison of overall characteristics across databases.

Description	WoSCC	Scopus	WoSCC + Scopus
Timespan	2006:2025	2006:2025	2006:2025
Sources (Journals, Books, etc)	481	838	970
Documents	1374	2255	2659
Annual Growth Rate %	7.63	6.53	6.93
Document Average Age	8.41	7.9	8
Average citations per doc	50.37	48.54	49.33
References	64731	15083	74991
Keywords Plus (ID)	2915	14961	15063
Author’s Keywords (DE)	2243	3911	4412
Authors	6914	10581	12939
Authors of single-authored docs	49	133	157
Single-authored docs	64	152	183
Co-Authors per Doc	6.35	5.94	5.91
International co-authorships %	18.7	18.31	18.2
article	892	1348	1595
review	482	907	1064

### 3.2 Publication trends and geographic distribution

**Figure 2:**
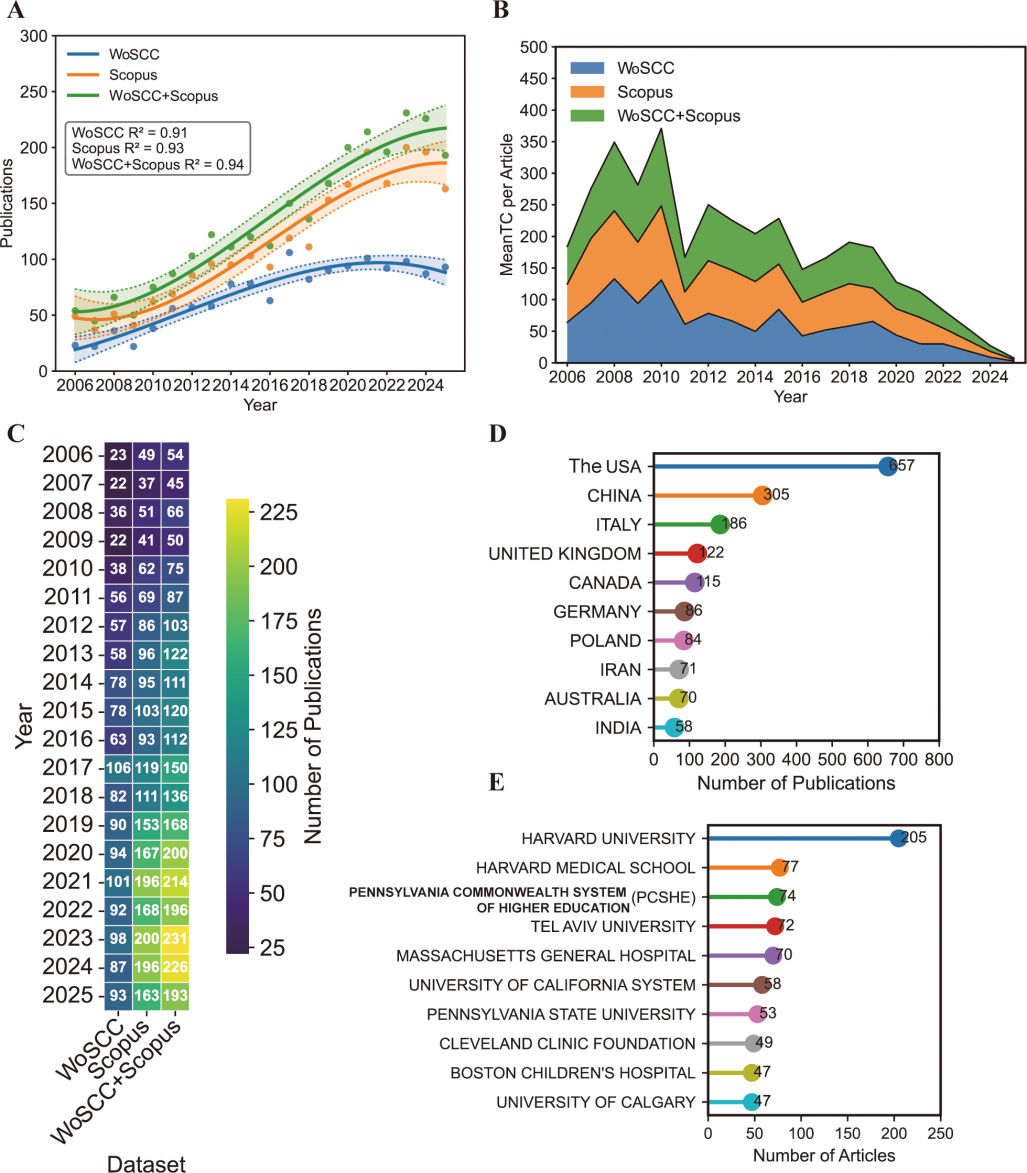
Annual publication trends and geographic distribution of vitamin D research in IBD (2006–2025). **(A)** Annual publication counts show an accelerated growth trajectory. **(B)** Yearly citation counts from WoSCC and Scopus, with peaks in 2008 and 2010. **(C)** Heatmap showing rapid growth from 2020 to 2024, peaking at 231 publications in 2023. **(D)** Top ten countries by publication volume. **(E)** Top ten institutions by publication volume.

The annual publication trend and the fitting curve results from the cubic polynomial regression model based on Ordinary Least Squares (OLS) revealed that the coefficient of determination (R²) for all three datasets exceeded 0.9. This indicates an overall acceleration in the number of publications in the field of vitamin D and IBD research from 2006 to 2025 (**Figure 2A**). Notably, the publication rate increased more rapidly after 2014, suggesting a sustained rise in attention and growing research interest in this area. **Figure 2B** and **Supplementary Table 2** illustrate the annual citation changes for each database. The results show that the citation frequency of articles in this field reached relatively high levels in 2008 (108.44 citations) and 2010 (122.25 citations). Based on the fitting curve results, we further conducted a heatmap analysis for each data point in **Figure 2A** (**Figure 2C**). The results indicate that the overall annual publication volume in this field exhibited a gradual increasing trend over the years, with a particularly rapid growth period from 2020 to 2024, peaking in 2023 (231 articles). Analysis of author countries revealed that the USA had the most publications (657, 24.7%), followed by CHINA (305, 11.5%), Italy (186, 7%), and the UK (122, 4.6%). Furthermore, among the top ten countries by number of publications, Iran (32.4%), Germany (27.9%), and India (27.6%) showed relatively high Multiple Country Publication (MCP) ratios, suggesting stronger international academic collaboration and interaction in these countries (**Figure 2D**; **Table 2**). Furthermore, the supplementary quantitative analysis of PubMed also showed that the USA ranked first in the number of related publications. This result was generally consistent with the main trends observed in WoSCC and Scopus, further supporting the stability of the bibliometric findings of this study (**Supplementary Table 3**). Harvard University and its affiliated medical school led in institutional output with 282 articles, followed by the Pennsylvania Commonwealth System of Higher Education (74). U.S. institutions dominated the top ten list, with only Tel Aviv University (Israel) and the University of Calgary (Canada) as non-U.S. entities, indicating a high concentration of academic output (**Figure 2E**).

**Table 2:** Top ten publishing countries and their collaboration rates.

Country	Articles	Articles %	SCP	MCP	MCP %
The USA	657	24.7	554	103	15.7
CHINA	305	11.5	281	24	7.9
ITALY	186	7	153	33	17.7
UNITED KINGDOM	122	4.6	89	33	27
CANADA	115	4.3	85	30	26.1
GERMANY	86	3.2	62	24	27.9
POLAND	84	3.2	76	8	9.5
IRAN	71	2.7	48	23	32.4
AUSTRALIA	70	2.6	57	13	18.6
INDIA	58	2.2	42	16	27.6

### 3.3 Analysis of author, institutional, and national productivity

**Figure 3:**
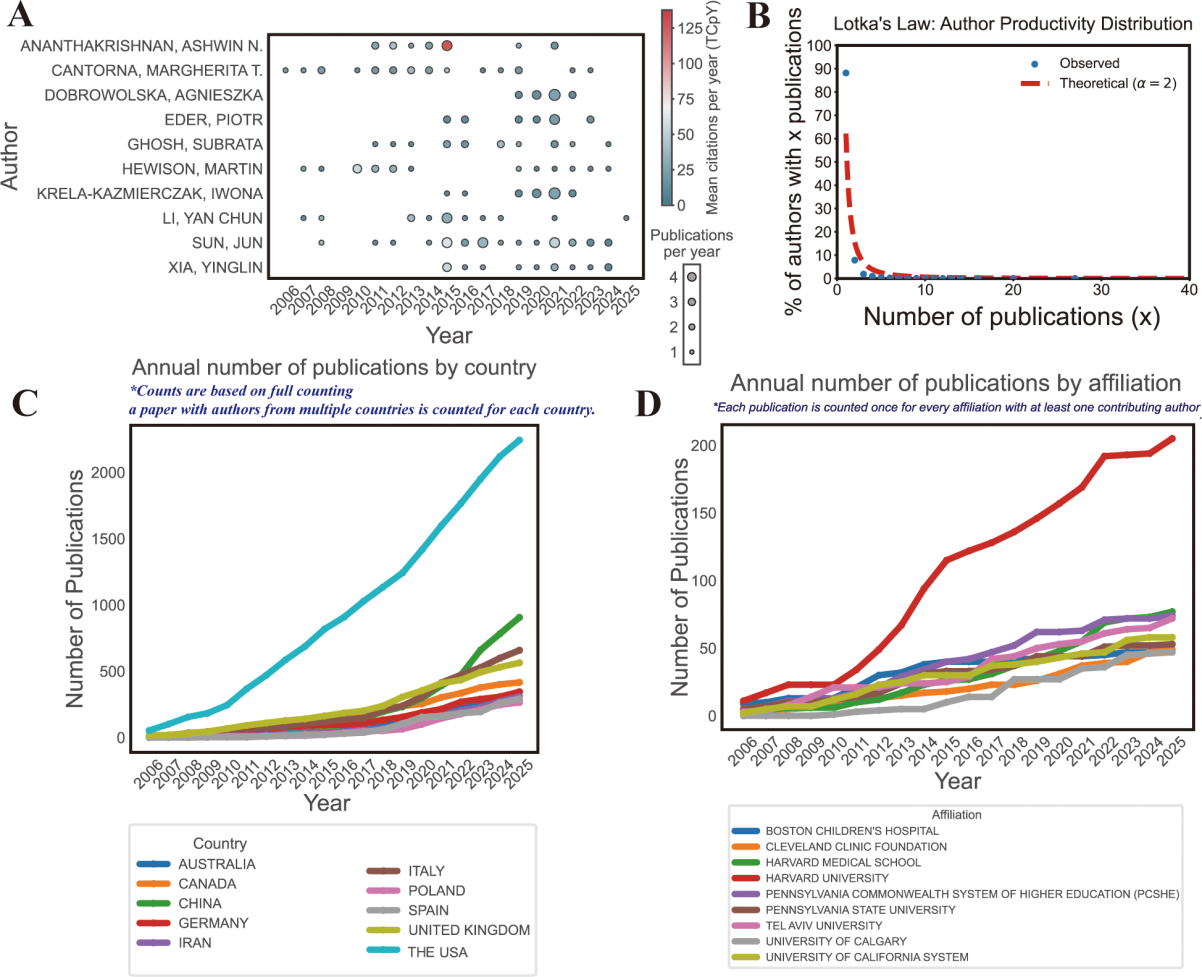
Productivity analysis of authors, countries, and institutions. **(A)** Top ten authors ranked by publication count and citation frequency.**(B)** Distribution of author productivity follows Lotka’s law. **(C)** Annual publication trends by country. **(D)** Annual publication trends by institution.

A total of 12,939 authors participated in this field of research. **Table 3** presents the top 10 authors ranked by H-index and displays their corresponding index values. Among them, Sun J has the highest number of publications (n = 27), followed by Cantorna M.T. (n = 20), Hewison M. (n = 16), Ananthakrishnan A.N. (n = 14), and Li YC (n = 14). In terms of citation impact, Ananthakrishnan A.N. (n = 3,209), Sun J (n = 2,698), and Cantorna M.T. (n = 2,661) were the leading contributors, demonstrating strong academic influence in this research area. An author’s publication count and average annual citations reflect their research activity and academic contribution to some extent. Notably, Ananthakrishnan A.N. achieved the highest average annual citation count (TCpy > 100) for a paper published in 2015, indicating strong academic dissemination. Based on the screening results for growth magnitude within the time window, authors such as Sun J and Xia YL show an upward trend in both publication count and citation numbers, indicating that the participation and contributions of Chinese scholars of vitamin D in IBD research are continuously increasing (**Figure 3A**). As shown in **Figure 3B**, 10% of authors have published more than 3 papers, while 90% have published only 1, suggesting that the distribution of author productivity in this field follows Lotka’s law. As publication years increase, the annual publication output from different countries shows a sustained growth trend. Notably, the USA has published 2,200 papers by 2025, far more than any other country, underscoring its dominance and contribution in this field (**Figure 3C**). At the institutional level, as each research institution’s focus on vitamin D and IBD continues to grow, its annual publication output generally exhibits a year−by−year upward trend. Among them, the number of publications from Harvard University increased from fewer than 20 in 2006 to approximately 200 in 2025, reflecting sustained growth in research output and its long-standing leadership in this field of study (**Figure 3D**).

**Table 3:** Ranking of the top ten authors based on academic metrics.

Author	h_index	g_index	m_index	TC	NP	PY_start
SUN JUN	21	27	1.105	2698	27	2008
CANTORNA MARGHERITA T.	18	20	0.857	2661	20	2006
ANANTHAKRISHNAN ASHWIN N.	13	14	0.813	3209	14	2011
LI YAN CHUN	12	14	0.6	1798	14	2007
GHOSH SUBRATA	11	14	0.688	1064	14	2011
HEWISON MARTIN	11	16	0.55	2498	16	2007
LU RONG	11	11	0.733	1099	11	2012
O’SULLIVAN MARIA	11	11	0.524	638	11	2006
XIA YINGLIN	11	13	0.917	1162	13	2015
GUBATAN JOHN	10	12	1	618	12	2017

### 3.4 Keyword analysis

**Figure 4:**
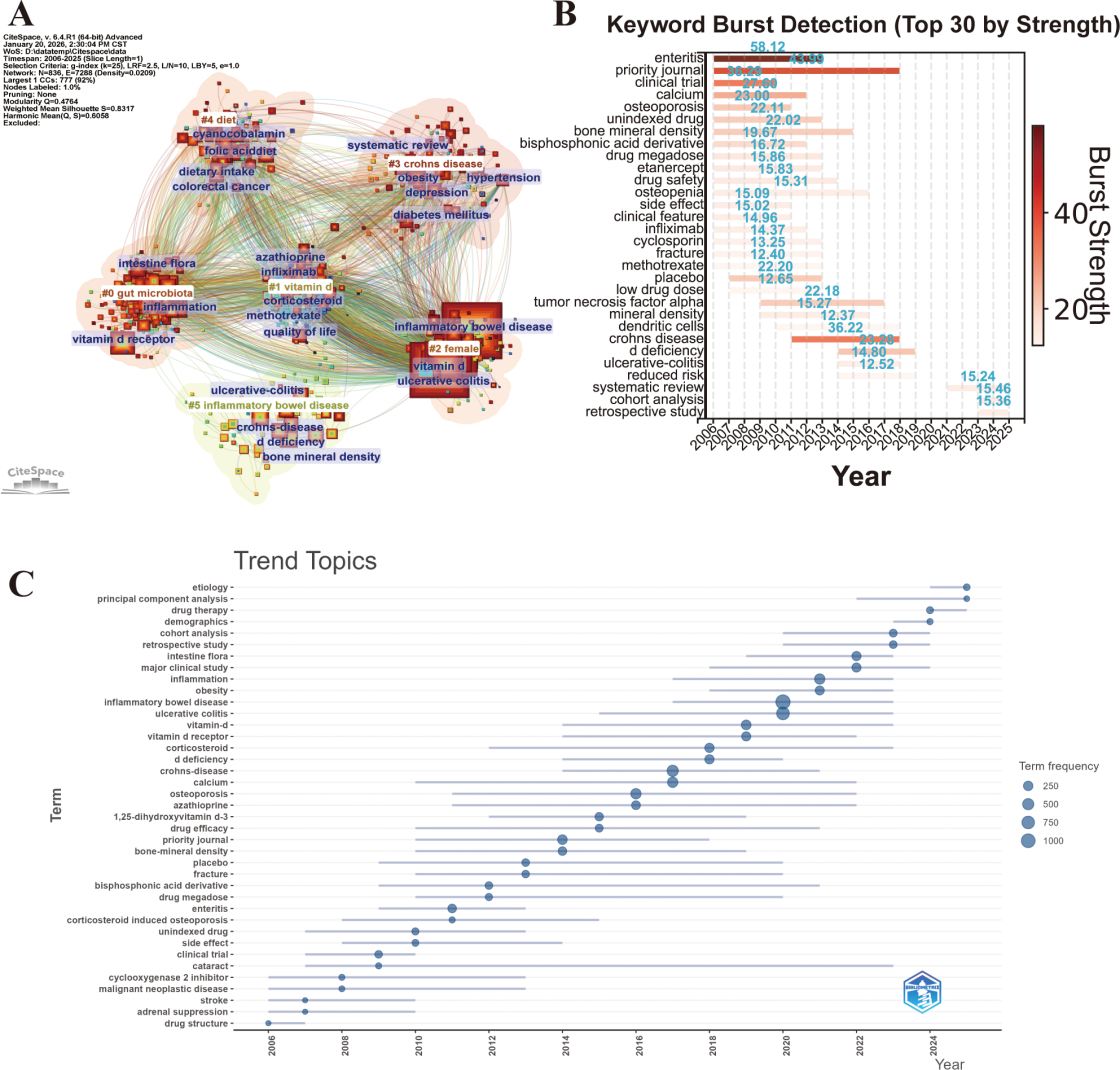
Keyword co-occurrence, clustering, and burst analysis. **(A)** Network of high-frequency keyword clusters. **(B)** Top 30 keywords with the strongest citation bursts. **(C)** Temporal dynamics of keyword thematic evolution.

Keyword clustering analysis is crucial for quickly understanding the core themes and hotspots in a research field. In our study, **Supplementary Table 4** lists the top 20 high-frequency keywords (≥ 198). Among them, the keyword with the highest frequency is inflammatory bowel disease (n = 1,801), followed by vitamin D (n = 1,462), Crohn disease (n = 1,420), ulcerative colitis (n = 1,054), vitamin D deficiency (n = 697), and calcium (n = 354). Using CiteSpace for keyword clustering analysis, the analysis reveals 6 thematic clusters that form a structurally clear, tightly connected network (**Figure 4A**). These clusters include gut microbiota, vitamin D, female, Crohn’s disease, diet, and inflammatory bowel disease. Researchers within these clusters focus on gut microbiota, VDR signaling, immune-inflammatory regulation, female populations, Crohn’s disease, chronic conditions such as obesity, hypertension, and depression, dietary and nutritional factors, and bone metabolism alterations associated with vitamin D deficiency. This pattern indicates that vitamin D serves as a potential research focus linking gut microbiota, immune regulation, and complications in IBD. Specifically, the #0 gut microbiota is closely associated with keywords such as inflammation, intestinal flora, and vitamin D receptor, indicating that the gut microecology and its related inflammatory mechanisms have become a research hotspot in this field. #1 The vitamin D primarily revolves around keywords such as quality of life, corticosteroid, methotrexate, and infliximab, indicating that vitamin D continues to receive significant attention in the clinical management of IBD. #5 centered on inflammatory bowel disease, forms strong connections with ulcerative colitis, Crohn’s disease, vitamin D deficiency, and bone mineral density. This suggests that vitamin D deficiency can contribute to the development of IBD, and also increases the risk of reduced bone mineral density and osteoporosis in IBD patients. Overall, keywords including vitamin D, gut microbiota, IBD, Crohn’s disease, and diet are distributed across different clusters. The interconnections among these clusters collectively reveal the field’s core research directions.

To accurately identify the evolving research hotspots and frontier directions in the field of vitamin D and IBD, we employed CiteSpace software to identify the 30 keywords with the highest citation bursts and visualized them using Python (**Figure 4B**). The results show that from 2006 to 2016, the burst keywords were primarily concentrated on the basic characteristics, pathological mechanisms, and treatment of the disease, including enteritis, clinical trial, Crohn’s disease, priority journal, drug megadose, calcium, and bone mineral density. Among these, enteritis (Burst: 58.12) and priority journal (Burst: 43.99) exhibited the highest burst strengths. Since 2020, keywords such as systematic review, cohort analysis, and retrospective study have emerged, indicating a gradual evolution of the research field toward evidence-based clinical approaches such as systematic reviews and cohort studies.

This study utilized Bibliometrix to generate a dynamic thematic evolution map of keywords, further predicting future research trends in the field of vitamin D and IBD. Such maps effectively track the temporal development of research topics within a field, clearly illustrating the evolution of research hotspots. From 2006 to 2010, the research focus was primarily on drug structure and indicators of adverse effects, with key changes centered on terms such as drug structure, cyclooxygenase-2 inhibitor, and adrenal suppression. During 2011-2017, themes such as osteoporosis, vitamin D deficiency, calcium, and bisphosphonic acid derivative frequently emerged, indicating increased attention to osteoporosis and nutritional metabolic abnormalities in IBD patients. Vitamin D deficiency and reduced calcium uptake were identified as potential key intervention targets. In recent years, the research direction has become more refined, shifting the focus to inflammatory bowel disease, ulcerative colitis, intestinal flora, obesity, and inflammation. This trend suggests a growing recognition of the role of immune regulation in this field. Furthermore, the increased frequency of keywords such as etiology, principal component analysis, demographics, and major clinical study reflects a shift from mechanistic exploration toward the development of high-quality evidence-based medicine (**Figure 4C**).

### 3.5 Collaboration among authors, countries, and institutions

**Figure 5:**
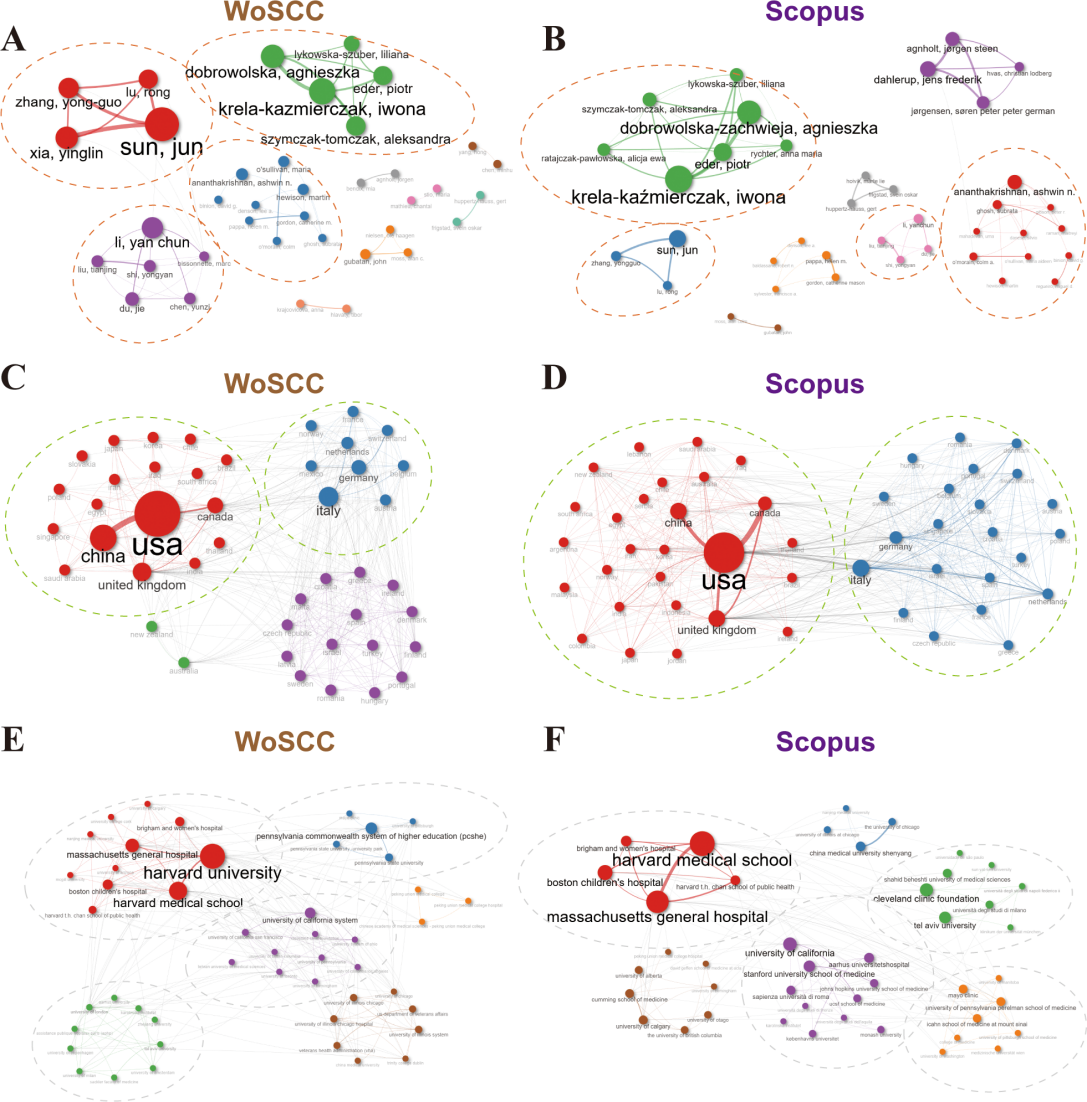
Multidimensional bibliometric comparison of WoSCC and Scopus databases. **(A)** Author collaboration network in the WoSCC database. **(B)** Author collaboration network in the Scopus database. **(C)** Country collaboration network in the WoSCC database. **(D)** Country collaboration network in the Scopus database. **(E)** Institutional collaboration network in the WoSCC database. **(F)** Institutional collaboration network in the Scopus database.

To evaluate the reliability and consistency of the WoSCC and Scopus databases in mapping author, country, and institutional collaboration networks, we used VOSviewer to perform a visualization analysis of leading authors, countries, and institutions. This analysis validated the consistency of the two databases in revealing the structure of collaborative networks. In this section, the main contributors were described using predefined bibliometric features in the collaboration maps. These features included node size, cluster position, network centrality, and total link strength. This criterion prevented selective emphasis on any specific country, author, or institution (**Figures 5A–F**). In the author collaboration networks, both databases revealed several closely linked clusters, indicating the presence of main research teams in the field. Clusters centered around Dobrowolska A., Krela-Kazmierczak I., and Szymczak-Tomczak A. appeared in both networks and occupied prominent central positions, reflecting robust collaborative networks. Chinese scholars centered on Sun J, Zhang YG, and Liu R, as well as international scholars centered on Ananthakrishnan A.N., Gordon C.M., and O’Sullivan M., demonstrated strong linkage intensity in both data repositories. However, the collaborative strength of the author cluster centered on Sun J, Zhang YG, Liu R, and Xia YL was relatively weaker in the Scopus database. While the two databases are consistent in identifying the primary author collaboration clusters, the subtle differences highlight the significant value of integrating multi-source data repositories. Additionally, two databases place the USA at the core, forming a tightly connected red cluster with the UK and Canada, showing its major role in international collaboration. The European country collaboration cluster centered around Italy and Germany was also identified in both databases. However, in the Scopus data repository, although the USA remains dominant, its link strength with CHINA is relatively weak, while its connections with the UK and Canada are stronger. Regarding institutional collaboration, both databases highlight a tightly connected institutional network centered on Harvard University, Harvard Medical School, and Massachusetts General Hospital, indicating their influence in this research field. Compared to the WoSCC database, Scopus also highlights the strengthened linkages between CHINA Medical University and the University of Chicago, suggesting relatively active collaboration among transnational institutions in this area. Although minor differences exist between the two databases, the core collaborative network clusters remain largely consistent. This indicates that an integrative analysis of the two databases further improves the reliability and coverage of the results, providing a more complete understanding of global research on vitamin D in IBD.

### 3.6 Citation analysis

The literature in this field was published across 970 journals. Among these, NUTRIENTS recorded the most publications (n = 141, Total citations = 8,025). This was followed by INFLAMMATORY BOWEL DISEASES (n=90, Total citations = 5,970), FRONTIERS IN IMMUNOLOGY (n = 56, Total citations = 3,645), and WORLD JOURNAL OF GASTROENTEROLOGY (n = 55, Total citations = 4,226). In terms of H-index, both INFLAMMATORY BOWEL DISEASES (H-index = 44) and NUTRIENTS (H-index = 44) emerged as the most academically influential journals in this field (**Supplementary Table 5**).

**Figure 6:**
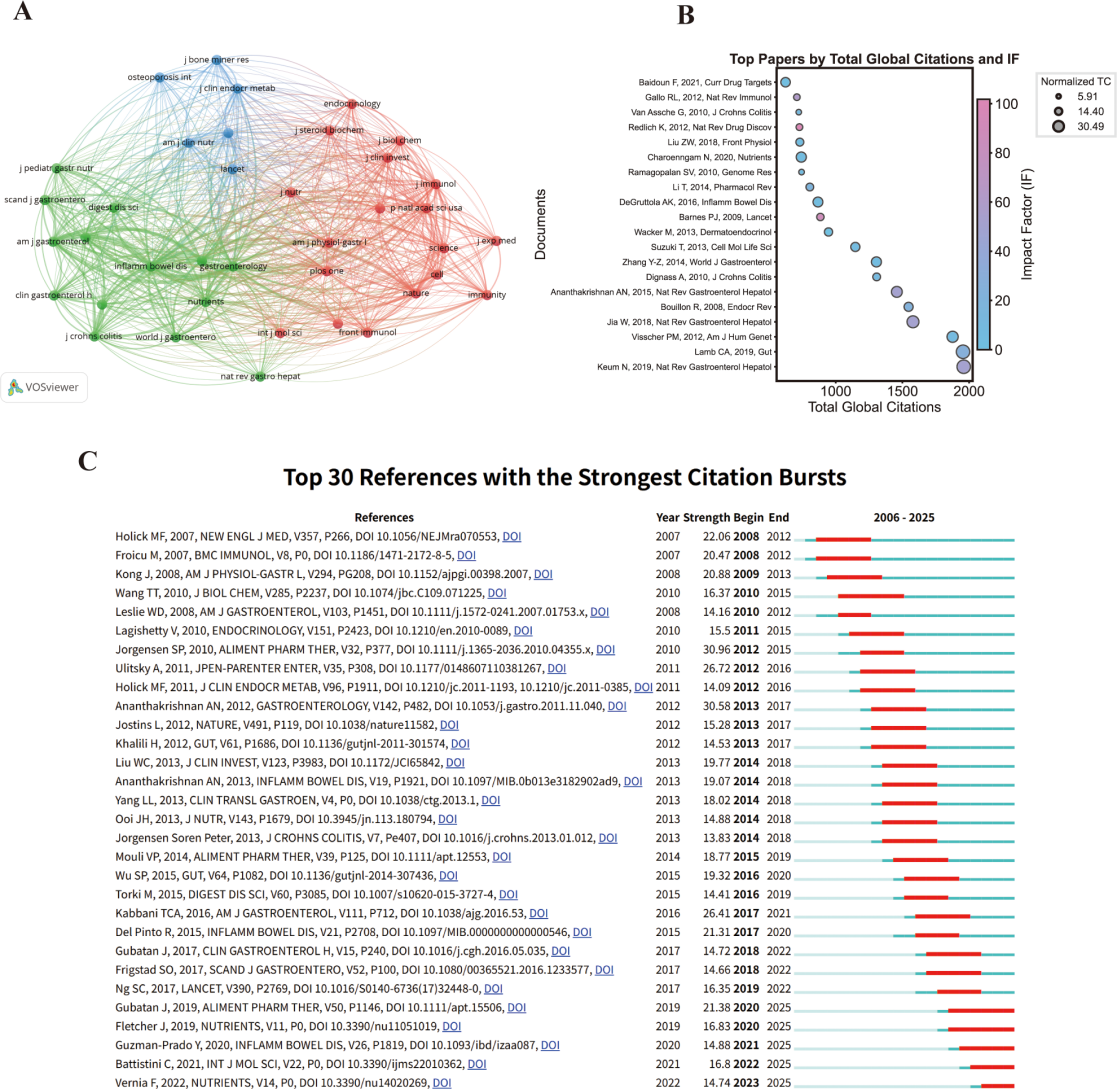
Citation analysis. **(A)** Co-cited journal network. Green clusters are centered on Inflamm Bowel Dis, Gastroenterology, World J Gastroenterol, and Nutrients, whereas red clusters include Nature, Science, Cell, Immunity, J Exp Med, and Front Immunol. **(B)** Bubble chart of the top 20 globally cited publications with corresponding journal impact factors. **(C)** Top 30 references ranked by citation burst strength.

We further used VOSviewer software to construct a journal co-citation visualization network, which was divided into three clusters (**Figure 6A**). Specifically, the green cluster was centered on Inflamm Bowel Dis (n = 4,239, TLS = 219,169), Gastroenterology (n = 4,174, TLS = 202,356), World J Gastroenterol (n = 1,459, TLS = 62,771), and Nutrients (n = 1,693, TLS = 80,867). In contrast, the red cluster primarily included Nature (n = 1,615, TLS = 112,043), Science (n = 1,033, TLS = 72,661), Immunity, J Exp Med, and Front Immunol (n = 1,660, TLS = 9,1002). Meanwhile, the blue cluster was represented by J Clin Endocr Metab (n = 1,287, TLS = 59,681), J Bone Miner Res, and Osteoporos Int (**Supplementary Table 6**). Notably, the three clusters are closely interconnected, which indicates that the research field of vitamin D and IBD exhibits characteristics of multidisciplinary integration and development. Building upon these findings, this study also employed a dual-map overlay analysis to further visualize the distribution, citation patterns, and evolving research foci within publications on vitamin D and IBD. In this map, the colored lines show citation links, with citing journals on the left and cited journals on the right. The results show that publications in the Molecular/Biology/Genetics domain are frequently cited by journals in the Medicine/Medical/Clinical and Molecular/Biology/Immunology fields. Likewise, publications in the Health/Nursing/Medicine domain are often cited by journals in Medicine/Medical/Clinical and Molecular/Biology/Immunology (**Supplementary Figure 3**).

We identified the 20 most cited papers in the vitamin D and IBD research using the Bibliometrix package (**Table 4**). The most highly cited papers included Keum N, 2019, Nat Rev Gastroenterol Hepatol (Total Citations=1959, Normalized TC = 30.49, IF = 50.9), Lamb CA, 2019, Gut (Total Citations=1954, Normalized TC=,30.41, IF = 25.8), Visscher PM, 2012, Am J Hum Genet (Total Citations=1876, Normalized TC = 21.21, IF = 8.1), Jia W, 2018, Nat Rev Gastroenterol Hepatol (Total Citations=1579, Normalized TC = 24.113, IF = 50.9), Bouillon R, 2008, Endocr Rev (Total Citations=1546, Normalized TC = 14.26, IF = 21.9). These highly cited publications, which were concentrated between 2008 and 2019, primarily addressed clinical management of IBD and its associated conditions, the gut microbiota, cancer risk, and the role of vitamin D. For example, Lamb CA et al. characterized IBD as a heterogeneous, chronic inflammatory disorder of the gastrointestinal tract that demands coordinated treatment. Clinicians deliver pharmacotherapy, nutritional support, surgery, and psychological care tailored to each patient’s individual needs. Bouillon et al. observed that vitamin D and its receptor not only maintain calcium–phosphate and bone metabolic homeostasis but also influence immune response, cancer progression, cardiovascular health, and metabolic functions. Analysis of total citations and journal impact factors indicated that these highly cited publications are predominantly published in influential journals. This finding highlights the authority and scholarly influence of research in this field (**Figure 6B**).

**Table 4:** Top twenty globally cited publications with detailed information.

Paper	DOI	Total citations	Normalized TC	IF
Keum N, 2019, Nat Rev Gastroenterol Hepatol	https://www.doi.org/10.1038/s41575-019-0189-8	1959	30.49	50.9
Lamb CA, 2019, Gut	https://www.doi.org/10.1136/gutjnl-2019-318484	1954	30.41	25.8
Visscher PM, 2012, Am J Hum Genet	https://www.doi.org/10.1016/j.ajhg.2011.11.029	1876	21.21	8.1
Jia W, 2018, Nat Rev Gastroenterol Hepatol	https://www.doi.org/10.1038/nrgastro.2017.119	1579	24.11	50.9
Bouillon R, 2008, Endocr Rev	https://www.doi.org/10.1210/er.2008-0004	1546	14.26	21.9
Ananthakrishnan AN, 2015, Nat Rev Gastroenterol Hepatol	https://www.doi.org/10.1038/nrgastro.2015.34	1458	20.22	50.9
Dignass A, 2010, J Crohns Colitis	https://www.doi.org/10.1016/j.crohns.2009.12.002	1307	10.69	8.7
Zhang Y-Z, 2014, World J Gastroenterol	https://www.doi.org/10.3748/wjg.v20.i1.91	1305	17.30	5.4
Suzuki T, 2013, Cell Mol Life Sci	https://www.doi.org/10.1007/s00018-012-1070-x	1147	14.53	6.2
Wacker M, 2013, Dermatoendocrinol	https://www.doi.org/10.4161/derm.24494	946	11.99	0
Barnes PJ, 2009, Lancet	https://www.doi.org/10.1016/S0140-6736(09)60326-3	885	9.80	88.5
DeGruttola AK, 2016, Inflamm Bowel Dis	https://www.doi.org/10.1097/MIB.0000000000000750	867	16.76	4.2
Li T, 2014, Pharmacol Rev	https://www.doi.org/10.1124/pr.113.008201	806	10.68	17.2
Ramagopalan SV, 2010, Genome Res	https://www.doi.org/10.1101/gr.107920.110	744	6.09	5.5
Charoenngam N, 2020, Nutrients	https://www.doi.org/10.3390/nu12072097	742	17.65	5
Liu ZW, 2018, Front Physiol	https://www.doi.org/10.3389/fphys.2018.00477	731	11.16	3.4
Redlich K, 2012, Nat Rev Drug Discov	https://www.doi.org/10.1038/nrd3669	728	8.23	101.8
Van Assche G, 2010, J Crohns Colitis	https://www.doi.org/10.1016/j.crohns.2009.09.009	723	5.91	8.7
Gallo RL, 2012, Nat Rev Immunol	https://www.doi.org/10.1038/nri3228	709	8.01	60.8
Baidoun F, 2021, Curr Drug Targets	https://www.doi.org/10.2174/1389450121999201117115717	624	15.43	2.5

To accurately identify the research hotspots and frontiers in the field of vitamin D and IBD, this study used CiteSpace software to detect the top 30 articles with the strongest citation bursts (**Figure 6C**). The top 3 with the highest burst strengths were: ① Clinical trial: vitamin D3 treatment in Crohn’s disease: a randomized double-blind placebo-controlled study (Burst strength = 30.96). ② Higher predicted vitamin D status is associated with reduced risk of Crohn’s disease (Burst strength = 30.58). ③ Vitamin D deficiency in patients with inflammatory bowel disease: association with disease activity and quality of life (Burst strength = 18.24).

### 3.7 Thematic evolution analysis

**Figure 7:**
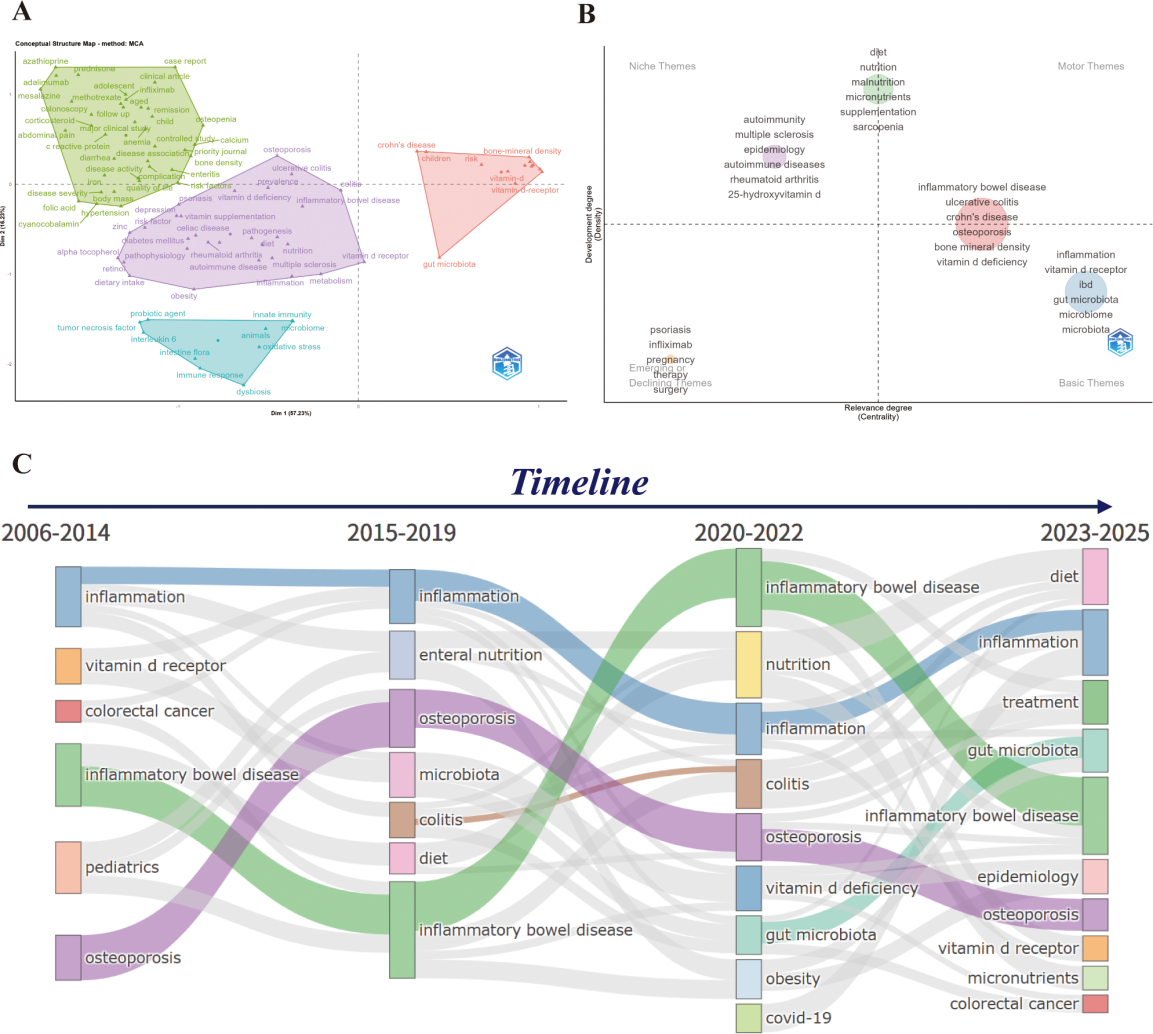
Thematic evolution analysis. **(A)** Multiple correspondence analysis (MCA). Purple clusters represent themes centered on IBD, ulcerative colitis, autoimmune disease, vitamin D deficiency, and nutrition. Blue clusters reflect evolving interactions between gut microbiota and the immune system. Term proximity indicates semantic similarity, whereas distant terms represent distinct conceptual directions. **(B)** Theme centrality analysis. The x-axis shows citation-based centrality, reflecting core significance in the network; the y-axis shows theme breadth, representing the range of related keywords. **(C)** Dynamic map of research theme evolution over time.

Multiple Correspondence Analysis (MCA) helps researchers identify hotspots and evolutionary trends within a research field by analyzing the interrelationships among research topics. Four main clusters were identified (**Figure 7A**). The green cluster mainly included drug treatments (azathioprine, prednisone, adalimumab, methotrexate), related complications (anemia, osteopenia, psoriasis, depression), and quality-of-life measures, showing a focus on the clinical management of IBD. The purple cluster contained terms such as IBD, UC, autoimmune disease, vitamin D deficiency, and nutrition, indicating a strong association between low vitamin D levels and IBD. The blue cluster focused on intestinal flora, dysbiosis, microbiome, and probiotics, highlighting the key role of gut microbiota and its interaction with the immune system. The red cluster centered on Crohn’s disease, vitamin D, gut microbiota, and bone mineral density indicates that vitamin D deficiency may negatively affect bone density in IBD patients and points to vitamin D as a potential treatment target, especially in children with intestinal disorders.

To further broaden the scope of analysis, this study used the Bibliometrix package to construct a thematic evolution strategic diagram (**Figure 7B**). The basic themes include inflammation, vitamin D receptor, IBD, gut microbiota, microbiome, and microbiota. They show high relevance but relatively low developmental maturity. In contrast, the motor themes linked to IBD and bone metabolism, including vitamin D deficiency and bone mineral density, are mature topics and have strong relevance in this field. Diet, nutrition, malnutrition, micronutrients, supplementation, and sarcopenia were classified as themes with high development but moderate relevance, suggesting that dietary interventions and micronutrient supplementation in IBD have emerged as relatively stable areas of investigation. Finally, emerging or declining themes such as psoriasis, infliximab, pregnancy, therapy, and surgery showed low development and relevance. This is likely due to limited high-quality evidence, stemming from new targeted therapies and ethical constraints. As a result, research in these areas remains exploratory.

Topic dynamic evolution analysis can reveal the dynamic evolution process of research themes in a specific field (**Figure 7C**). In this study, from 2006 to 2014, the research primarily focused on themes such as inflammation, IBD, vitamin D receptor, and osteoporosis, indicating that early research emphasized the pathological mechanisms of IBD inflammation and the related functions of vitamin D. From 2015 to 2019, the research focus gradually expanded from purely inflammatory mechanisms to the field of nutritional immunology, encompassing enteral nutrition, microbiota, diet, and colitis, suggesting a growing interest in the roles of diet, nutrition, and gut microecology in IBD. From 2020 to 2022, the intensity of core themes, including inflammatory bowel disease, inflammation, nutrition, colitis, and osteoporosis, remained unchanged, indicating that the association between inflammatory mechanisms and nutritional metabolism in IBD remains a key research focus. However, the emergence of new hotspots such as COVID-19, obesity, and gut microbiota suggests the potential roles of gut microbiota, obesity-related metabolic mechanisms and viral infection on the effects of immune modulation by vitamin D in IBD. From 2023 to 2025, the research focus gradually shifted toward treatment, epidemiology, and micronutrients, suggesting that clinical translation may become the primary goal in this field, with micronutrients and dietary therapy emerging as effective treatment approaches for inflammatory bowel disease.

### 3.8 Topic trend analysis based on the BERTopic model

**Figure 8:**
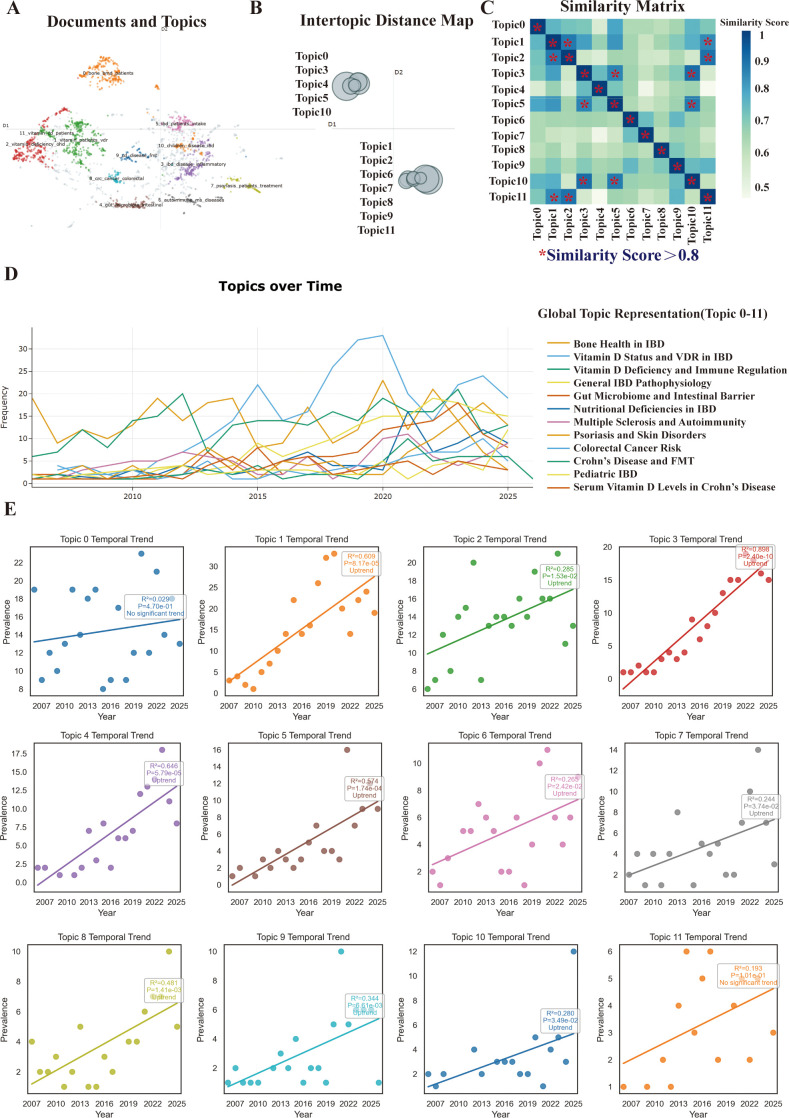
BERTopic model analysis of thematic trends. **(A)** Document–topic projection, with each dot representing a publication classified by topic label. **(B)** Topic distribution map illustrating intrinsic correlations among topics. Topics 0, 3, 4, and 5 cluster near Topic 10, whereas Topics 1, 2, 6, 7, 8, and 9 form tightly connected clusters with Topic 11. **(C)** Topic similarity matrix showing high similarity scores between Topics 3, 5, and 10, and between Topics 1, 2, and 11. **(D)** Temporal evolution of BERTopic themes, highlighting emerging research hotspots. **(E)** Linear regression analysis of individual topic trends over time, with R² values used to classify temporal patterns.

To more accurately analyze research frontiers and evolutionary pathways of vitamin D in IBD, this study used Python to perform topic modeling on 2,659 articles. In **Figure 8A**, each scattered point represents an article, classified by different Topic labels. The analysis identified 12 Topics, which showed good spatial separation. We further distinguished these topics using PCA (**Figure 8B**). The results show that Topics 0: Bone Health in IBD, 3: General IBD Pathophysiology, 4: Gut Microbiome and Intestinal Barrier, 5: Nutritional Deficiencies in IBD, and 10: Pediatric IBD are more closely distributed. This suggests a strong correlation among these themes. At the same time, Topic 1: Vitamin D Status and VDR in IBD, Topic 2: Vitamin D Deficiency and Immune Regulation, Topic 6: Multiple Sclerosis and Autoimmunity, Topic 7: Psoriasis and Skin Disorders, Topic 8: Colorectal Cancer Risk, Topic 9: Crohn’s Disease and FMT, and Topic 11: Serum Vitamin D Levels in Crohn’s Disease form a tightly connected cluster. Furthermore, a correlation matrix analysis of the 12 topics (**Figure 8C**) showed that General IBD Pathophysiology, Nutritional Deficiencies in IBD, and Pediatric IBD had similarity scores > 0.8, indicating a close relationship among the pathological mechanisms of IBD, nutrient deficiencies, and pediatric IBD patients. Research similarity was also high among Vitamin D Status and VDR in IBD, Serum Vitamin D Levels in Crohn’s Disease, and Vitamin D Deficiency and Immune Regulation. This suggests that vitamin D and its receptor-mediated immunomodulation may be key factors of IBD.

We applied BERTopic to identify 12 latent topics from a large text corpus. Topic labels were determined following manual review of the top 10 weighted terms and selected documents for each topic (**Supplementary Table 7**). The results show that topics with a larger volume of literature included Topic1: Vitamin D Status and VDR in IBD (n = 288), Topic3: General IBD Pathophysiology (n = 164), followed by Topic4: Gut Microbiome and Intestinal Barrier (n = 123) and Topic5: Nutritional Deficiencies in IBD (n = 97). To visualize the temporal dynamics of these topics, we plotted line graphs showing the frequency of each topic’s occurrence over publication years (**Figure 8D**). Starting in 2015, research on Topic 1: Vitamin D Status and VDR in IBD showed steady growth, peaking in 2020, indicating that vitamin D and its receptor as a therapeutic target in IBD have received sustained attention. In recent years, Topics 4: Gut Microbiome and Intestinal Barrier and 9: Crohn’s Disease and FMT have shown an upward trend, indicating that research on the gut microbiome, intestinal barrier function, and fecal microbiota transplant is becoming an emerging hotspot in this field. Furthermore, this study used linear regression for each topic and classified trends based on R² and statistical significance (*P* < 0.01). The results show that topics with a significantly positive trend include Topic 1 (Vitamin D Status and VDR in IBD; R² = 0.609, *P* < 0.01), Topic 3 (General IBD Pathophysiology; R² = 0.898, *P* < 0.01), Topic 4 (Gut Microbiome and Intestinal Barrier; R² = 0.646, *P < 0.01*), Topic 5 (Nutritional Deficiencies in IBD; R² = 0.574, *P < 0.01*), Topic 8 (Colorectal Cancer Risk; R² = 0.481, *P < 0.01*), and Topic 9 (Crohn’s Disease and FMT; R² = 0.344, *P < 0.01*) (**Figure 8E**; **Supplementary Table 8**). These results indicate that the importance of topics such as vitamin D and its receptor, the fundamental mechanism of IBD, the gut microbiome, nutrient deficiencies, colorectal cancer risk, and fecal microbiota transplant continues to rise.

## 4 Discussion

### 4.1 Current status of vitamin D in IBD based on bibliometric analysis

This study conducted a bibliometric analysis of 2,659 publications on vitamin D in the treatment of IBD published between January 1, 2006, and December 31, 2025. These publications comprised 1,595 articles and 1,064 reviews. Our findings indicate that research on vitamin D in IBD has made substantial progress. Since 2014, the annual number of publications in this field has increased rapidly, with the period from 2020 to 2024 marking an accelerated phase of development. This study also demonstrates a high degree of interdisciplinary collaboration. Extensive collaborative networks among authors, countries, and institutions provide strong support for the further development of this field. Notably, the USA and Harvard University, Harvard Medical School occupy prominent positions in the research, suggesting that they have contributed substantially to global collaboration. This pattern may be closely related to the large population of patients with IBD, the substantial disease burden, and the relatively abundant research resources in the USA (19). Keywords and cluster analysis identified that vitamin D deficiency, calcium, bone mineral density, gut microbiota, and diet were key themes. Thematic evolution analysis and BERTopic modeling revealed recent growth in the gut microbiome, intestinal barrier, nutritional deficiencies, Crohn’s disease, and fecal microbiota transplantation (FMT). The emergence of keywords such as intestinal barrier function and FMT underscores the growing importance of gut microbiota and immune regulation in IBD pathogenesis and treatment. Strategies such as micronutrients and dietary therapy have become research hotspots, highlighting the role of nutritional immunology in disease management. Keyword evolution shows a shift from basic mechanistic studies to higher-quality translational research, with systematic reviews, cohort studies, and retrospective studies now supporting clinical applications.

### 4.2 Randomized controlled trials of vitamin D in IBD

Since 2020, keywords such as systematic review, cohort analysis, and retrospective study have emerged. This trend suggests that research on vitamin D and IBD is gradually moving from mechanistic exploration toward clinical validation. To further supplement the evidence from clinical trials and address the limited scope of clinical studies in WoSCC and Scopus, we analyzed relevant RCTs in PubMed. Vitamin D is a low-cost therapy that may reduce the chance of adverse outcomes in IBD (20). Its clinical potential has become increasingly evident as research progresses from preclinical studies to clinical trials. Analysis of randomized controlled trials on vitamin D and IBD (**Table 5**) reveals common processes through which vitamin D improves IBD symptoms. Whether in children (21) or adult patients (22), during active UC (23) or quiescent CD (24), supplementation with vitamin D via oral administration (25) or intramuscular injection (26) safely and effectively increases the levels of 25(OH)D in IBD patients, with good tolerability. However, minor differences remain in the specific protocols. Regarding dosing, studies indicate that daily oral Vitamin D at higher doses is more effective than lower-dose regimens at increasing 25(OH)D levels. Specifically, daily oral intake of 2,000 IU vitamin D3 is superior to daily oral intake of vitamin D2 (27). Further research has found that a weight-based vitamin D dosing regimen is not more effective than plan with a fixed amount (28). In terms of dosing frequency, some studies suggest that a “stoss therapy” of vitamin D may have clinical effects comparable to weekly dosing regimens (29). Notably, one study showed that although continuous supplementation with vitamin D3 for 6 weeks improved 25(OH)D levels at week 8, this effect disappeared by the 12-week follow-up (30). Therefore, further research is needed to refine the dosing and administration frequency regimens to achieve long-term maintenance of normal vitamin D status. Regarding molecular mechanisms, Regarding molecular mechanisms, vitamin D reduces the expression of inflammatory factors, inhibiting Th1-type immune responses, and attenuates the pro-angiogenic response in UC patients with vitamin D deficiency (31–33).

**Table 5:** Analyses of randomized controlled trials on vitamin D and IBD.

Study	Population	Intervention	Control	Outcomes	Key findings
Rose Lee 2020	children patients with IBD and hypovitaminosis D	Stoss therapy Vitamin D3 300,000 IU/once	Vitamin D3 50,000 IU/once	25(OH)D; calcium; PTH	Stoss therapy safely and effectively raises serum 25(OH)D levels in pediatric patients with inflammatory bowel disease, producing effects comparable to weekly supplementation regimens.
Doaa El Amrousy 2021 (21)	children patients with IBD and hypovitaminosis D	Vitamin D3 2,000 IU/d	Placebo	QOL; inflammatory markers; the safety of vitamin D	Vitamin D supplementation may confer beneficial effects in children with inflammatory bowel disease.
Jonathan EM O’Donnell 2025 (29)	Children patients with IBD and hypovitaminosis D	Stoss therapy Vitamin D3 3–12 age: 400,000 IU/once >12 age: 800,000 IU/once	Vitamin D3 2,000 IU/d	Biochemistry; stool markers; anthropometrics; clinical disease indices; medication	At 12 months, stoss therapy increased serum 25(OH)D levels to an extent comparable to daily supplementation with 2,000 IU of vitamin D3.
Helen M Pappa 2014	patients with IBD	VitaminD2 400 IU/d	Vitamin D2 Summer 1,000 IU/d winner 2,000 IU/d	25(OH)D; CRP; IL-6	Daily oral doses of up to 2,000 IU of vitamin D2 were insufficient to sustain optimal serum 25(OH)D levels; although they were well tolerated. In contrast, participants receiving higher doses of vitamin D2 exhibited a lower incidence of elevated inflammatory markers and cytokines.
Helen M Pappa 2012 (27)	children and adolescents patients with hypovitaminosis D	Vitamin D3–2000 IU/d VitaminD2 50,000 IU/week	Vitamin D2 2,000 IU/d	25(OH)D; PTH; the adverse event occurrence rate	Daily oral supplementation with 2,000 IU vitamin D3 and weekly administration of 50,000 IU vitamin D2 were more effective than daily 2,000 IU vitamin D2 in elevating serum 25(OH)D concentrations; and both regimens were well tolerated.
Robert Z Simek 2016 (30)	Children with IBD and hypovitaminosis D	Vitamin D3 10,000 IU/10 kg/week	Vitamin D3 5,000 IU/10 kg/week	25(OH)D; Ca; PTH	Both vitamin D supplementation regimens effectively increased serum 25(OH)D concentrations in children with IBD at week 8. However, this effect was no longer observed by week 12, underscoring the need for ongoing supplementation to maintain long-term optimal vitamin D status.
Kirstin E Wingate 2014	Children with quiescent CD	Vitamin D3 2,000 IU/d	Vitamin D3 400 IU/d	25(OH)D	Daily supplementation with 2,000 IU vitamin D3 more effectively increased serum 25(OH)D concentrations compared with 400 IU. However, both doses were comparable in achieving 25(OH)D thresholds of 16 ng/mL or 20 ng/mL.
Bei Tan 2018 (22)	IBD and hypovitaminosis D	Vitamin D3 150,000 IU/once/3 months Calcium 200 mg/tid/d	Calcium 200 mg/tid/d Vehicle	25(OH)D; BMD; disease activity	Vitamin D supplementation is essential for the management of patients with IBD who are vitamin D deficient.
Rizwan Ahamed Z 2019 (23)	active UC and hypovitaminosis D	Vitamin D3 60,000 IU/d	Placebo	UCDAI scores; CRP; ESR; fecal calprotectin	Oral vitamin D3 supplementation was associated with reduced disease activity and severity; particularly among patients with active UC who attained target serum vitamin D levels of 40 ng/mL.
Sara Karimi 2019	regimens in UC patients with hypovitaminosis D	Vitamin D 2,000 IU/d	Vitamin D 12,000 IU/d	25(OH)D; TAC; TOS; SCCAI score;IBDQ-9 score	Daily supplementation with 2,000 IU vitamin D effectively increased serum 25(OH)D concentrations, improved quality of life, and reduced disease activity in patients with UC and concomitant vitamin D deficiency.
Sara Karimi 2020 (25)	mild to moderate active UC patients	Vitamin D 2,000 IU/d	Vitamin D 1,000 IU/d	inflammatory biomarkers; disease activity; quality of life; anthropometric indices; dietary intakes; physical activity	Daily supplementation with 2,000 IU vitamin D for 12 weeks effectively prevented systemic inflammation and reduced disease activity in patients with mild-to-moderate active UC.
Amrollah Sharifi 2020 (26)	mild-to-moderate UC patients	Vitamin D3 a single muscular injection 7.5 mg	Placebo	CD40L	Vitamin D supplementation in patients with mild-to-moderate UC induced downregulation of the CD40L gene, a critical mediator of inflammatory signaling pathways.
Amrollah Sharifi 2019 (31)	mild-to-moderate UC patients	Vitamin D3 a single muscular injection 7.5 mg	Placebo	IL-4; IL-10; IL-12p70; IFN-γ; TNF-α	Vitamin D may selectively suppress Th1 immune responses while leaving Th2 responses unaffected.
Mohammad Reza Emami 2020 (32)	patients with UC	Vitamin D a single intramuscular injection of 300,000 IU	Placebo	25(OH)D; Visfatin; VEGF	Vitamin D supplementation may reduce the expression of pro-angiogenic factors in UC patients with low serum 25(OH)D levels.
Amrollah Sharifi 2016 (24)	UC in remission	Vitamin D3 300,000 IU intramuscular	Placebo	25 (OH); PTH; Calcium; hCAP/LL37; ESR; hs-CRP;	Vitamin D3 supplementation decreased ESR and hs-CRP levels while increasing LL-37 gene expression in patients with UC.
Amrollah Sharifi 2018	mild to moderate UC patients	Vitamin D3 300,000 IU a single injection	Placebo	BDI score;25(OH)D	Elevated serum vitamin D levels may exert antidepressant effects.
SP Jørgensen 2010	CD in remission	Vitamin D3 1,200 IU/d	Placebo	25(OH)D; relapse rate	Daily oral supplementation with 1,200 IU vitamin D3 markedly increased serum 25(OH)D levels.
Neeraj Narula 2016	CD in remission	Vitamin D3 10,000 IU/d	Vitamin D3 1,000 IU/d	25(OH)D; relapse rate; mood scores	Daily high-dose oral supplementation with 10,000 IU vitamin D3 markedly increased serum 25(OH)D levels.
Naziha Berriche-Yahi 2022 (33)	CD patients with hypovitaminosis D	Vitamin D3 6,000 IU/d	Vitamin D3 200,000 IU/month	25(OH)D; CDAI score; fecal calprotectin;pro-inflammatory cytokines; trace elements;antioxidant status	Vitamin D supplementation reduced serum inflammatory markers; corrected nutritional deficiencies; and alleviated CD, with continuous daily administration of 6,000 IU demonstrating greater efficacy than intermittent monthly doses of 200,000 IU.
Vladimir Kojecky 2020 (28)	IBD	Vitamin D3 28 IU/kg/d	Vitamin D3 2,000 IU/d	25(OH)D	Weight-adjusted vitamin D supplementation offered no advantage over fixed-dose regimens in maintaining stable serum 25(OH)D levels in patients with IBD, and daily administration of 2,000 IU cholecalciferol was safe and sufficient during the winter season.
Fiona O’Sullivan 2019	CD in remission	Vitamin D3 2,000 IU/d	Placebo	25(OH)D	Exposure to sunlight represents a key factor regulating circulating 25(OH)D concentrations, thereby substantially impacting vitamin D status and immune function.

### 4.3 Core and emerging themes in research on vitamin D and IBD

#### 4.3.1 Core themes

##### 4.3.1.1 Bone health

According to analyses of keyword co-occurrence, clustering, and thematic evolution, bone health has emerged as a relatively well-established theme in research on vitamin D and IBD. Chronic intestinal inflammation can induce the release of inflammatory cytokines, thereby promoting osteoclastogenesis and accelerating bone resorption (34). It can also impair osteoblast function and disrupt the dynamic balance between bone resorption and bone formation (35). Vitamin D deficiency is an important factor associated with abnormal bone metabolism in IBD. It can reduce osteoblast activity, suppress bone formation, and increase fracture risk (36). Vitamin D and its receptor activate the TRPV6, enhancing Ca²^+^ uptake and absorption (37). Activation of the VDR may also promote RANKL expression (38). This participates in osteoclastogenesis and releases calcium stored in bone into the circulation (39). Glucocorticoids are potent anti-inflammatory agents that are effective in treating IBD. However, their long-term use may increase apoptosis, reduce osteoblast formation, and increase osteoclast numbers (40). Existing evidence suggests that vitamin D supplementation may improve biomechanical properties, promote fracture callus remodeling, and help prevent fracture risk. To delay progression of bone loss, early vitamin D supplementation at 800–1,000 IU/day is recommended for patients with IBD (41). Therefore, future studies should focus not only on the effect of vitamin D on 25(OH)D but also on its long-term effects on bone mineral density and fracture risk.

##### 4.3.1.2 Gut microbiota

This study found that gut microbiota was closely associated with inflammation, intestinal flora, and the vitamin D receptor. The microbiome, microbiota, and intestinal barrier have gradually emerged. Previous studies mainly focused on vitamin D deficiency and inflammatory responses. Recent research has increasingly focused on interactions among the gut microbiota and immune regulation (42). Previous studies have shown that vitamin D deficiency can lead to gut microbial dysbiosis and increase susceptibility to intestinal injury. The abundance of pathogenic bacteria is increased. Meanwhile, beneficial bacteria are reduced in patients with IBD. These changes suggest that gut microbial imbalance may further aggravate intestinal inflammation (43, 44). VDR deficiency can reduce E-cadherin expression in intestinal epithelial cells and decrease the number of tolerogenic dendritic cells. This induces gut dysbiosis and intestinal inflammation (45). Because bacterial microbiota do not express VDR, vitamin D may maintain gut microbial homeostasis through bacterial products or VDR signaling pathways in intestinal epithelial cells and immune cells (46). Therefore, the interaction between vitamin D and gut microbiota may be an important direction for future IBD research. This direction clarifies how vitamin D influences intestinal inflammation by integrating the regulation of microbial balance, barrier integrity, and mucosal immune responses.

##### 4.3.1.3 Diet and micronutrients

The thematic evolution analysis showed that the research focus gradually expanded during 2015–2019. The research focus gradually expanded from inflammation to nutrition and the immune system. For example, keywords such as diet, nutrition, malnutrition, micronutrients, supplementation, and sarcopenia showed high development and moderate relevance. This indicates that dietary intervention and micronutrient supplementation have gradually become stable research topics in this field (47). Dietary management in IBD is not limited to adjusting nutritional requirements but is also used as an adjunctive strategy to control disease activity (48). Notably, diet has a substantial influence on the gut microbiota (49). Consequently, fresh fruits and vegetables, monounsaturated fats, complex carbohydrates, and lean proteins are important dietary strategies for IBD management. The NHS recommends oily fish, such as salmon and sardines, as important dietary sources of vitamin D. Liver, red meat, egg yolks, breakfast cereals, and fortified fat spreads may also contribute to adequate vitamin D intake (50). At the same time, it should be noted that IBD patients are generally advised to avoid vegetables, wheat products, dairy products, and processed meat products (51). Vitamin D2 and Vitamin D3 are common oral vitamin D supplements. Both are metabolized in the liver to 25(OH)D, but vitamin D3 is more effective than vitamin D2 in increasing 25(OH)D levels (52). The National Institute for Health and Care Excellence recommends a daily intake of 400 IU of vitamin D (53), the Institute of Medicine recommends 600 IU/day to maintain serum 25(OH)D levels above 50 nmol/L (54), and the Endocrine Society recommends 1,500–2,000 IU/day for adults (55). Recent studies have suggested that patients with IBD may require daily doses ranging from 1,800 IU to 10,000 IU (56). These evolving recommendations demonstrate that research on vitamin D and IBD is gradually moving beyond single nutrient supplementation toward a broader framework that integrates diet, micronutrients, and the gut microbiota.

#### 4.3.2 Emerging themes

##### 4.3.2.1 Probiotics

The MCA analysis showed that the cluster was mainly centered on intestinal flora, dysbiosis, microbiome, and probiotic agents, reflecting the important role of interactions between the gut microbiota and the immune system in this field. The emergence of probiotics indicates that attention has been increasing to the synergistic effects of vitamin D with microbiota-modulating strategies. Probiotics can directly replenish beneficial bacteria depleted in patients with IBD and inhibit the growth and proliferation of harmful bacteria. They may also reduce direct contact between bacteria and epithelial cells (57). In addition, probiotics can enhance the expression of epithelial tight junction proteins, reduce intestinal permeability, and prevent the invasion or translocation of toxins and bacteria (58). Some probiotics may also inhibit histamine receptor activity and reduce inflammatory responses (59). Previous studies have shown that the combined use of vitamin D and probiotics may exert synergistic protective effects in IBD by promoting VDR expression in intestinal epithelial cells (60). Probiotics can regulate gene expression related to vitamin D metabolism in the gut and improve intestinal vitamin D absorption (61). They may also activate VDR in intestinal cells, enhance the anti-inflammatory effects, and indirectly reduce intestinal inflammation (62). Therefore, future research on vitamin D and IBD may gradually shift from single-nutrient supplementation toward an integrated intervention of vitamin D and probiotics.

##### 4.3.2.2 Obesity

**Figure 9:**
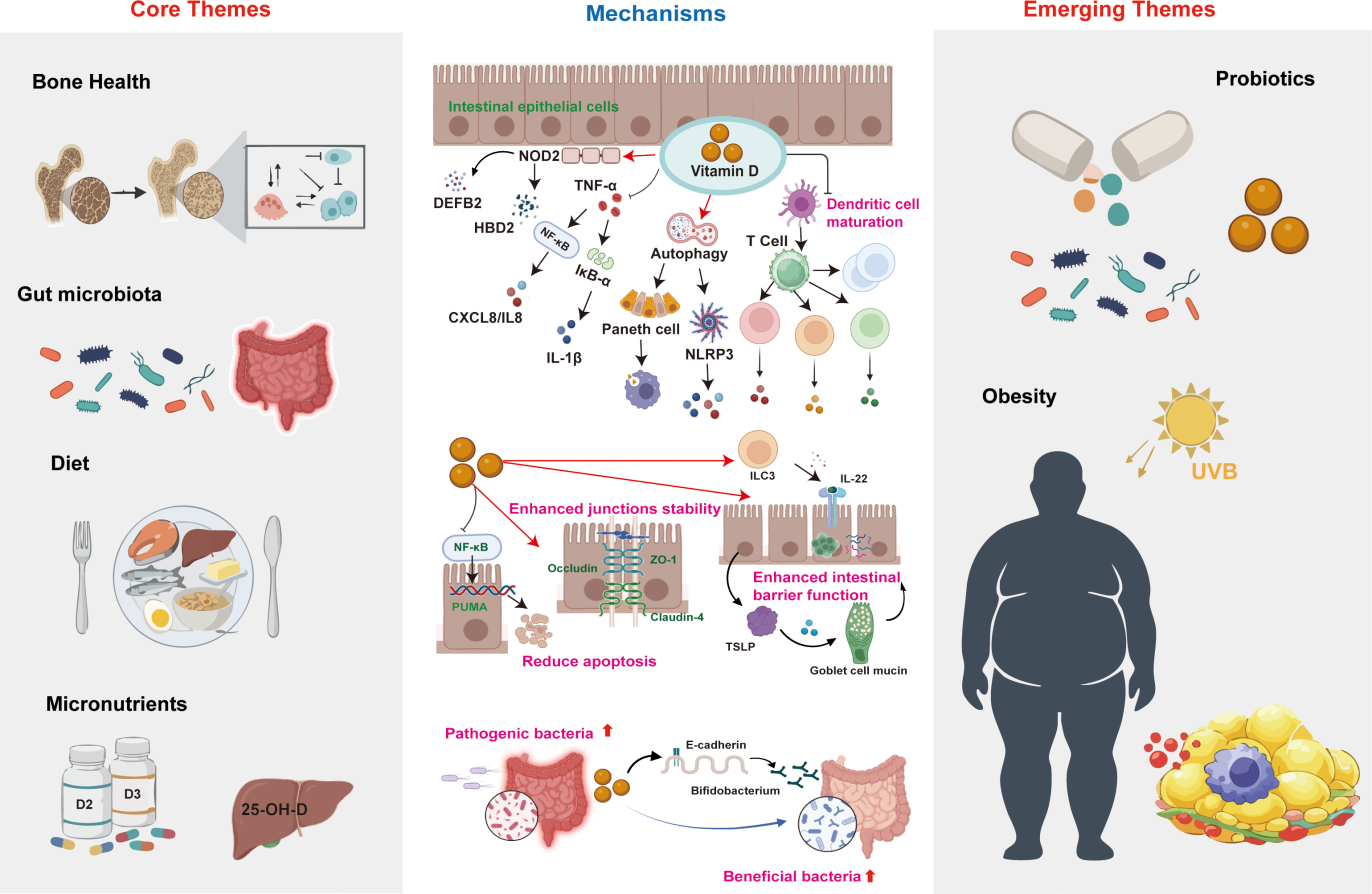
Potential mechanisms, core topics, and emerging directions in vitamin D and IBD.

Obesity emerged as one of the newly identified keywords after 2020, a trend consistent with changes in the global epidemiology of IBD. Obesity is not only an important risk factor for vitamin D deficiency but also a chronic inflammatory state (63). Patients with obesity are generally at increased risk of vitamin D deficiency due to adipose tissue sequestration of vitamin D and insufficient sunlight exposure. Clinical studies have shown that vitamin D supplementation in obesity may provide multiple health benefits (64). It can increase anti-inflammatory adipokines and reduce pro-inflammatory cytokines (65). It may also promote insulin secretion from pancreatic β cells, increase adiponectin and IGF-1 secretion, reduce hepatic triglyceride accumulation (66), and prevent abnormal activation of RAS (67). Increased adipose tissue may act as a storage reservoir, trapping circulating vitamin D and thereby reducing its bioavailability (68). At the same time, vitamin D can affect the activity of deacetylases and AMPK in adipocytes (69). In addition, vitamin D is also involved in the secretion and regulation of various adipokines, which play important roles in food intake control, lipid and glucose metabolism, and insulin sensitivity (70) (**Figure 9**).

### 4.4 Topic-based discussion identified by BERTopic modeling

#### 4.4.1 Topics with sustained growth trends

We employed BERTopic to perform topic modeling analysis on a large text corpus and found that the importance of topics such as gut microbiota, fecal microbiota transplant, and nutritional deficiencies has been consistently increasing. A growing body of evidence indicates that the gut microbiota can regulate innate immune activation and maintain energy metabolism, immune homeostasis, and intestinal barrier integrity (71). For example, the gut microbiota metabolizes tryptophan into indole derivatives, which modulate intestinal inflammation via the AHR signaling pathway. Conversely, certain indole metabolites produced by bacteria may also induce DNA damage and increase intestinal permeability (72).

As an emerging therapeutic strategy, fecal microbiota transplant (FMT) can help relieve the clinical symptoms of IBD with a favorable safety profile (73). Its mechanisms of action primarily include correcting intestinal flora imbalance, regulating microbial metabolites, and promoting the repair of damaged intestinal barriers (74). For example, FMT can increase the levels of beneficial bacteria and reduce the levels of harmful bacteria, thereby improving the microbial imbalance in IBD (75). Concurrently, after FMT treatment, the production of SCFA by certain bacteria in the recipient’s intestine increases, thereby inducing Treg generation and suppressing the expression of pro-inflammatory factors (76). Studies have found that FMT has a more significant short-term effect for UC patients. In some UC patients, after FMT treatment, endoscopic examination shows mucosal healing, suggesting that FMT may repair damaged intestinal barriers (77). Furthermore, approximately 78% of patients exhibit micronutrient deficiency as early as the initial stages of the disease (78). Low levels of vitamins and minerals can lead to higher levels of potential pathogens, thereby promoting an intestinal inflammatory response (79). Therefore, oral nutritional supplements, vitamins, and enteral and parenteral nutrition can be important components of comprehensive IBD management.

#### 4.4.2 Potential topics

In contrast to research topics showing a significant upward trend, the research interest in the direction of CD and serum vitamin D levels has remained relatively stable. Patients with CD frequently show reduced vitamin D levels as a result of factors such as a monotonous diet, impaired gastrointestinal tract absorption, insufficient outdoor activity, and glucocorticoid use (80). Studies indicate that serum 25(OH)D levels are associated with a tendency for CD patients to be in the active phase of the disease (81). In the healthy population, the recommended serum 25(OH)D concentration is generally above 50 nmol/L. However, research on the optimal range of vitamin D levels in CD patients remains limited (82). We speculate that this phenomenon may be due to the fact that the association between serum vitamin D levels and CD disease activity has already reached a relatively clear consensus. However, this does not mean that this topic lacks research value. In the future, we should consider the influence of vitamin D receptor gene diversity and quantify its impact on key clinical prognostic outcomes in CD patients. Clarifying the dose-response relationship will help establish a unified recommended concentration for vitamin D management.

### 4.5 Limitations

This study has several methodological limitations that need careful consideration when interpreting the findings. First, only English-language publications were included to enhance consistency of citation data and allow cross-study comparisons. This strategy helped improve data standardization and international readability, but it may also have excluded studies published in non-English languages. Although such studies may have value within local academic contexts, their visibility and influence in the global citation network are generally limited. Second, the WoSCC and Scopus databases differ in coverage, update mechanisms, and document types. WoSCC offers a longer time span and a stable citation infrastructure. Scopus provides broader coverage and more frequent updates. This study used a cross-source deduplication strategy with field standardization to combine the strengths of both databases. This approach reduces systematic bias from differences between the databases. Differences in indexing rules and data structures may still affect the identification of institutional affiliations and the construction of collaboration networks. Standardizing author and institutional names is not always accurate with existing bibliometric tools, leading to occasional matching errors. Given the large sample size and long time frame, these technical deviations are not expected to alter overall trends or core conclusions.

It should also be noted that bibliometric analysis and topic modeling have inherent methodological limitations. For example, citation network structures can be influenced by citation bias. Highly cited publications may appear more central, while emerging studies with few citations may be underestimated. Co-word analysis depends on the standardization of author keywords and index terms. Variations in language or the use of synonyms may introduce bias when identifying topics. Results from cluster analysis and topic modeling are sensitive to threshold choices, algorithms, and parameter settings. Different methods may produce slightly different divisions of topic structures. Therefore, hotspot and topic evolution results should be seen as general trends, not definitive or exclusive classifications. Importantly, bibliometric analysis is macroscopic and retrospective. It is designed to outline the evolution and knowledge structure of a field, not to replace clinical investigations or mechanistic explorations. Within this framework, this study provides a quantitative reference for understanding the research landscape and trends in vitamin D research in IBD.

## 5 Conclusion

This analysis reveals rapid growth in research on vitamin D in IBD, highlighting the field’s interdisciplinary nature and its increasing translational medical relevance. Since 2014, the annual publication volume has continued to accelerate, reflecting the growing interest in the potential role of vitamin D in IBD management. Core research themes include vitamin D deficiency, gut microbiota, inflammatory responses, and dietary or nutritional interventions. Emerging research is increasingly focusing on the gut microbiome, immunomodulation, micronutrient interactions, and evidence generation and clinical translation. Existing mechanistic studies have proposed that vitamin D may be involved in immune regulation, intestinal barrier function, and gut microbial homeostasis. The findings provide a reference for future mechanistic studies and for generating clinical evidence in vitamin D-related IBD research.

## Data Availability

The original contributions presented in the study are included in the article/**Supplementary Material**. Further inquiries can be directed to the corresponding authors.
