# Supplementary material for: From nutritional intervention to immune modulation: a multi-database bibliometric and topic modeling study of vitamin D in inflammatory bowel disease
Source: Front Immunol. 2026 May 29;17:1845767. doi: 10.3389/fimmu.2026.1845767 (PMC13259713; doi:10.3389/fimmu.2026.1845767)
Supplement: Supplementary file 1 [file DataSheet1.pdf]

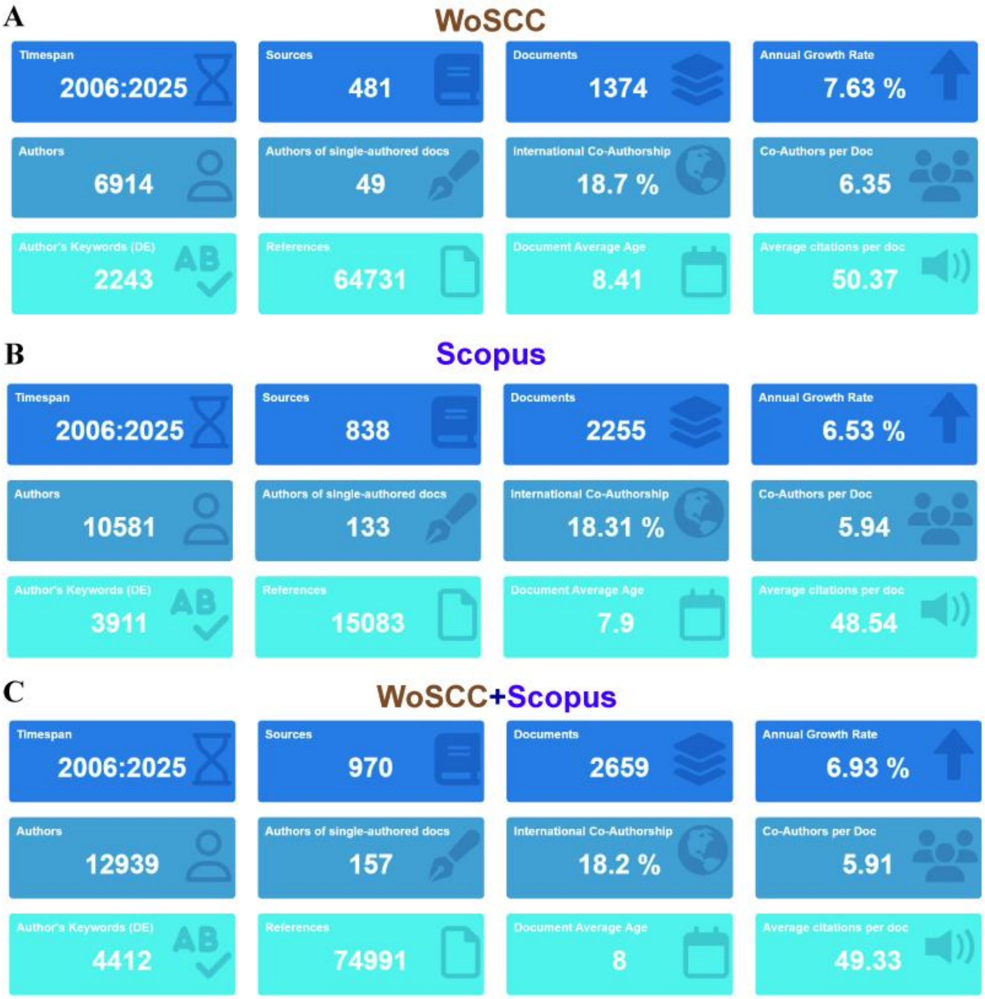

Supplementary Figure1. Comparative overview of databases. (A) Annual publication characteristics in WoSCC (2006–2025).(B) Annual publication characteristics in Scopus (2006–2025).(C) Comparative annual publication characteristics across WoSCC and Scopus databases.

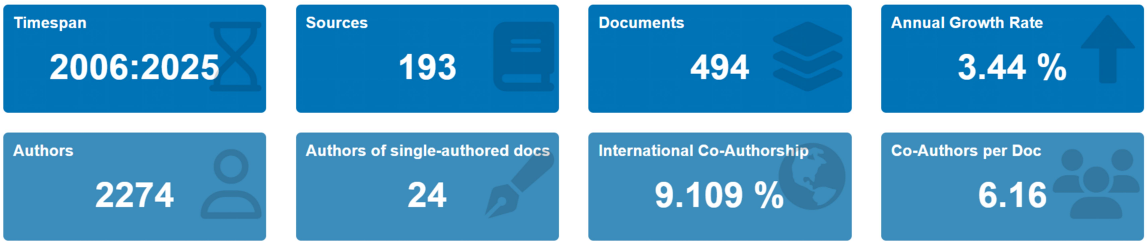

Supplementary Figure2. Main information of PubMed database

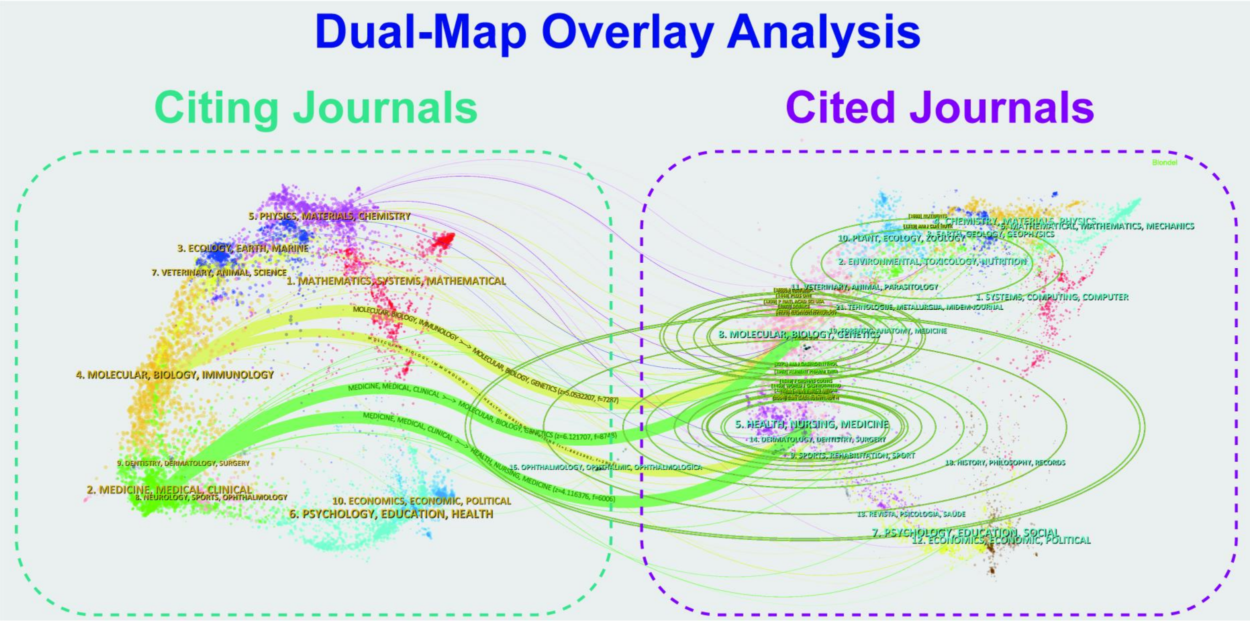

Supplementary Figure3. Dual-map overlay analysis depicting interdisciplinary citation flows. Colored trajectories indicate citation links from citing journals (left) to cited journals (right). Publications in Molecular/Biology/Genetics journals are primarily cited by journals in Medicine/Medical/Clinical and Molecular/Biology/Immunology, whereas publications in Health/Nursing/Medicine are cited across Medicine/Medical/Clinical and Molecular/Biology/Immunology.
