# Supplementary material for: From nutritional intervention to immune modulation: a multi-database bibliometric and topic modeling study of vitamin D in inflammatory bowel disease
Source: Front Immunol. 2026 May 29;17:1845767. doi: 10.3389/fimmu.2026.1845767 (PMC13259713; doi:10.3389/fimmu.2026.1845767)
Supplement: Supplementary file 2 [file DataSheet2.pdf]

**Supplementary Table1.** Literature search strategies for each database

| Database | Search Strategy | Search Date | Filtering Conditions |
|---|---|---|---|
| WoSCC | TS1=("Vitamin D" OR "Vit D" OR "Calcidiol" OR "Calcifediol" OR "Calciferol" OR "25OHD*");TS2=("inflammatory bowel disease" OR "IBD" OR "Crohn's disease" OR "ulcerative colitis");TS=TS1 AND TS2 | The literature search was conducted on January 16, 2026. | English-language publications from January 1, 2006, to December 31, 2025, including Articles and Review Articles. |
| Scopus | ( TITLE-ABS-KEY ( "Vitamin D" OR "Vit D" OR "Calcidiol" OR "Calcifediol" OR "Calciferol" OR "25OHD*" ) AND TITLE-ABS-KEY ( "inflammatory bowel disease" OR "IBD" OR "Crohn's disease" OR "ulcerative colitis" ) ) | The literature search was conducted on January 16, 2026. | English-language publications from January 1, 2006, to December 31, 2025, including Articles and Review Articles. |
| PubMed | (“Vitamin D”[MeSH] OR “Calcifediol”[MeSH]) AND (“Inflammatory Bowel Diseases”[MeSH] OR “Crohn Disease”[MeSH] OR “Colitis, Ulcerative”[MeSH]) | The literature search was conducted on January 16, 2026. | English-language publications from January 1, 2006, to December 31, 2025, including randomized controlled trial. |

**Supplementary Table2**. Comparison of annual citation trends across databases

| Year | WoSCC | Scopus | WoSCC+Scopus |
|---|---|---|---|
| 2006 | 64.09 | 60.57 | 59.57 |
| 2007 | 94.55 | 101.81 | 78.71 |
| 2008 | 132.83 | 107.80 | 108.44 |
| 2009 | 93.95 | 97.02 | 90.32 |
| 2010 | 130.82 | 117.73 | 122.25 |
| 2011 | 60.82 | 51.45 | 54.72 |
| 2012 | 78.26 | 83.37 | 88.47 |
| 2013 | 66.31 | 80.31 | 78.93 |
| 2014 | 49.58 | 79.09 | 75.44 |
| 2015 | 84.49 | 71.78 | 72.12 |
| 2016 | 42.59 | 53.40 | 51.74 |
| 2017 | 51.97 | 59.27 | 54.63 |
| 2018 | 58.30 | 66.92 | 65.49 |
| 2019 | 65.42 | 52.83 | 64.26 |
| 2020 | 43.87 | 41.69 | 42.03 |
| 2021 | 30.09 | 41.78 | 40.43 |
| 2022 | 29.90 | 25.07 | 28.03 |
| 2023 | 19.12 | 17.43 | 18.79 |
| 2024 | 8.71 | 9.39 | 8.87 |
| 2025 | 2.81 | 2.37 | 2.37 |

**Supplementary Table3.** Top ten countries and their collaboration rates in Pubmed

| Country | Articles | Articles % | SCP | MCP | MCP % |
|---|---|---|---|---|---|
| The USA | 80 | 16.2 | 77 | 3 | 3.8 |
| CHINA | 48 | 9.7 | 45 | 3 | 6.3 |
| CANADA | 27 | 5.5 | 26 | 1 | 3.7 |
| ITALY | 24 | 4.9 | 21 | 3 | 12.5 |
| AUSTRALIA | 18 | 3.6 | 14 | 4 | 22.2 |
| DENMARK | 17 | 3.4 | 14 | 3 | 17.6 |
| IRAN | 17 | 3.4 | 11 | 6 | 35.3 |
| POLAND | 13 | 2.6 | 13 | 0 | 0 |
| GERMANY | 11 | 2.2 | 8 | 3 | 27.3 |
| IRELAND | 11 | 2.2 | 10 | 1 | 9.1 |

**Supplementary Table4.** Top twenty keywords and their frequencies

| Keywords | Count |
|---|---|
| inflammatory bowel disease | 1801 |
| vitamin d | 1462 |
| crohn disease | 1420 |
| ulcerative colitis | 1054 |
| vitamin d deficiency | 697 |
| calcium | 354 |
| inflammatory bowel diseases | 323 |
| vitamin d receptor | 318 |
| osteoporosis | 294 |
| prevalence | 277 |
| risk factor | 275 |
| priority journal | 268 |
| inflammation | 252 |
| infliximab | 238 |
| corticosteroid | 227 |
| bone mineral density | 212 |
| intestine flora | 211 |
| major clinical study | 203 |
| controlled study | 202 |
| rheumatoid arthritis | 198 |

**Supplementary Table5.** Ranking of the top ten journals based on academic metrics

| Source | h_index | g_index | m_index | TC | NP | PY_start |
|---|---|---|---|---|---|---|
| INFLAMMATORY BOWEL DISEASES | 44 | 76 | 2.095 | 5970 | 90 | 2006 |
| NUTRIENTS | 44 | 87 | 2.75 | 8025 | 141 | 2011 |
| FRONTIERS IN IMMUNOLOGY | 31 | 56 | 2.214 | 3645 | 56 | 2013 |
| WORLD JOURNAL OF GASTROENTEROLOGY | 30 | 55 | 1.429 | 4226 | 55 | 2006 |
| JOURNAL OF CROHNS & COLITIS | 25 | 38 | 1.316 | 2149 | 38 | 2008 |
| INTERNATIONAL JOURNAL OF MOLECULAR SCIENCES | 20 | 44 | 1.333 | 1942 | 45 | 2012 |
| DIGESTIVE DISEASES AND SCIENCES | 18 | 38 | 0.947 | 1483 | 42 | 2008 |
| ALIMENTARY PHARMACOLOGY & THERAPEUTICS | 17 | 19 | 0.895 | 1620 | 19 | 2008 |
| JOURNAL OF PEDIATRIC GASTROENTEROLOGY AND NUTRITION | 16 | 34 | 0.762 | 1161 | 34 | 2006 |
| JOURNAL OF DIGESTIVE DISEASES | 13 | 17 | 0.813 | 655 | 17 | 2011 |

**Supplementary Table6.** Co-cited journal network: top twenty journals ranked by citation counts

| source | citations | total link strength |
|---|---|---|
| inflamm bowel dis | 4239 | 219169 |
| gastroenterology | 4174 | 202356 |
| gut | 3266 | 187946 |
| am j gastroenterol | 2271 | 116204 |
| aliment pharm ther | 1982 | 98253 |
| nutrients | 1693 | 80867 |
| j immunol | 1660 | 91002 |
| j crohns colitis | 1630 | 86555 |
| nature | 1615 | 112043 |
| world j gastroentero | 1459 | 62771 |
| plos one | 1444 | 79486 |
| am j clin nutr | 1321 | 64378 |
| j clin endocr metab | 1287 | 59681 |
| p natl acad sci usa | 1240 | 83781 |
| new engl j med | 1230 | 59116 |
| digest dis sci | 1209 | 58071 |
| lancet | 1071 | 54137 |
| j pediatr gastr nutr | 1048 | 51850 |
| science | 1033 | 72661 |
| clin gastroenterol h | 1005 | 52482 |

**Supplementary Table7.** Detailed information for each BERTopic theme label

| Topic | Count | Name | Representation |
|---|---|---|---|
| 0 | 289 | Bone Health in IBD | ['bone', 'bmd', 'patients', 'osteoporosis', 'density', 'ibd', 'risk', 'mineral', 'low', 'disease'] |
| 1 | 288 | Vitamin D Status and VDR in IBD | ['vitamin', 'patients', 'vdr', 'levels', 'colitis', 'ibd', 'mice', 'uc', 'disease', 'deficiency'] |
| 2 | 269 | Vitamin D Deficiency and Immune Regulation | ['vitamin', 'deficiency', 'ohd', 'diseases', 'immune', 'disease', 'autoimmune', 'role', 'studies', 'levels'] |
| 3 | 164 | General IBD Pathophysiology | ['ibd', 'disease', 'inflammatory', 'patients', 'bowel', 'intestinal', 'factors', 'treatment', 'chronic', 'gut'] |
| 4 | 123 | Gut Microbiome and Intestinal Barrier | ['gut', 'microbiota', 'intestinal', 'microbiome', 'immune', 'host', 'barrier', 'microbial', 'cells', 'human'] |
| 5 | 97 | Nutritional Deficiencies in IBD | ['ibd', 'patients', 'intake', 'nutritional', 'nutrition', 'dietary', 'micronutrient', 'deficiencies', 'disease', 'diet'] |
| 6 | 95 | Multiple Sclerosis and Autoimmunity | ['autoimmune', 'ms', 'diseases', 'genes', 'disease', 'genetic', 'disorders', 'thyroid', 'sclerosis', 'autophagy'] |
| 7 | 84 | Psoriasis and Skin Disorders | ['psoriasis', 'patients', 'treatment', 'skin', 'topical', 'psoriatic', 'hs', 'systemic', 'comorbidities', 'lesions'] |
| 8 | 69 | Colorectal Cancer Risk | ['crc', 'cancer', 'colorectal', 'risk', 'factors', 'chemoprevention', 'incidence', 'asperuloside', 'prevention', 'colon'] |
| 9 | 62 | Crohn’s Disease and FMT | ['cd', 'disease', 'fmt', 'patients', 'recurrence', 'crohns', 'cdi', 'infection', 'tuberculosis', 'treatment'] |
| 10 | 54 | Pediatric IBD | ['children', 'disease', 'ibd', 'patients', 'ulcerative', 'uc', 'colitis', 'crohns', 'pediatric', 'bowel'] |
| 11 | 50 | Serum Vitamin D Levels in Crohn’s Disease | ['vitamin', 'cd', 'patients', 'levels', 'serum', 'ngml', 'oh', 'ohd', 'crohns', 'disease'] |

**Supplementary Table8.** Linear regression statistics for BERTopic themes

| Topic | Linear_r2 | Linear_p |
|---|---|---|
| 0 | 0.029381 | 0.469937 |
| 1 | 0.608545 | 8.17E-05 |
| 2 | 0.285122 | 0.015307 |
| 3 | 0.897632 | 2.4E-10 |
| 4 | 0.646495 | 5.79E-05 |
| 5 | 0.57353 | 0.000174 |
| 6 | 0.264818 | 0.024172 |
| 7 | 0.243566 | 0.037399 |
| 8 | 0.481168 | 0.001409 |
| 9 | 0.343579 | 0.006606 |
| 10 | 0.28045 | 0.034885 |
| 11 | 0.193144 | 0.10119 |
